# Geometry, Allometry and Biomechanics of Fern Leaf Petioles: Their Significance for the Evolution of Functional and Ecological Diversity Within the Pteridaceae

**DOI:** 10.3389/fpls.2018.00197

**Published:** 2018-03-07

**Authors:** Jennifer N. Mahley, Jarmila Pittermann, Nick Rowe, Alex Baer, James E. Watkins, Eric Schuettpelz, James K. Wheeler, Klaus Mehltreter, Michael Windham, Weston Testo, James Beck

**Affiliations:** ^1^Department of Ecology and Evolutionary Biology, University of California, Santa Cruz, Santa Cruz, CA, United States; ^2^Botanique et Modélisation de l'Architecture des Plantes et des Végétations (AMAP) TA A-51/PS2, Montpellier, France; ^3^Department of Biology, California State University, Bakersfield, Bakersfield, CA, United States; ^4^Department of Biology, Colgate University, Hamilton, NY, United States; ^5^Department of Botany, National Museum of Natural History, Smithsonian Institution, Washington, DC, United States; ^6^Red de Ecología Funcional, Instituto de Ecología, Xalapa, Mexico; ^7^Department of Biology, Duke University, Durham, NC, United States; ^8^Pringle Herbarium, Department of Plant Biology, University of Vermont, Burlington, VT, United States; ^9^Biological Sciences, Wichita State University, Wichita, KS, United States

**Keywords:** sclerenchyma, ground tissue, flexural rigidity, modulus of elasticity, second moment of area

## Abstract

Herbaceous plants rely on a combination of turgor, ground tissues and geometry for mechanical support of leaves and stems. Unlike most angiosperms however, ferns employ a sub-dermal layer of fibers, known as a hypodermal sterome, for support of their leaves. The sterome is nearly ubiquitous in ferns, but nothing is known about its role in leaf biomechanics. The goal of this research was to characterize sterome attributes in ferns that experience a broad range of mechanical stresses, as imposed by their aquatic, xeric, epiphytic, and terrestrial niches. Members of the Pteridaceae meet this criteria well. The anatomical and functional morphometrics along with published values of tissue moduli were used to model petiole flexural rigidity and susceptibility to buckling in 20 species of the Pteridaceae. Strong allometric relationships were observed between sterome thickness and leaf size, with the sterome contributing over 97% to petiole flexural rigidity. Surprisingly, the small-statured cheilanthoid ferns allocated the highest fraction of their petiole to the sterome, while large leaves exploited aspects of geometry (second moment of area) to achieve bending resistance. This pattern also revealed an economy of function in which increasing sterome thickness was associated with decreasing fiber cell reinforcement, and fiber wall fraction. Lastly, strong petioles were associated with durable leaves, as approximated by specific leaf area. This study reveals meaningful patterns in fern leaf biomechanics that align with species leaf size, sterome attributes and life-history strategy.

## Introduction

Self-supporting plants should be tall enough to compete for light and to efficiently disperse seeds or spores, but not so tall as to cause instability or buckling. This balance has guided the evolution of plant form since the appearance of the earliest types of land plants, which like modern vegetation, had to contend with gravity, wind, rain and other disturbance (Niklas, 1992, 2004; Bateman et al., 1998). To this end, the mechanical stability in the early land plant *Aglaophyton major* from the Devonian, was achieved by turgor pressure in cortical parenchyma; turgor also supported *Rhynia* spp. and *Cooksonia*, two Devonian tracheophytes similar in size to *Aglaophyton*. As effective as turgor-based mechanics may have been for these plants, reliance on water for support restricted them to mesic habitats, capped their height at less than 40 cm and limited the lateral reach of their stems (Speck and Vogellehner, 1988, 1994; Bateman et al., 1998). The evolution of transport and support tissues such as secondary xylem and sclerenchyma solved these problems by releasing plants from their reliance on parenchymatous “hydrostats” for support, and allowing them to explore a greater diversity of habitats as well as a broader morphospace.

**Figure 1:**
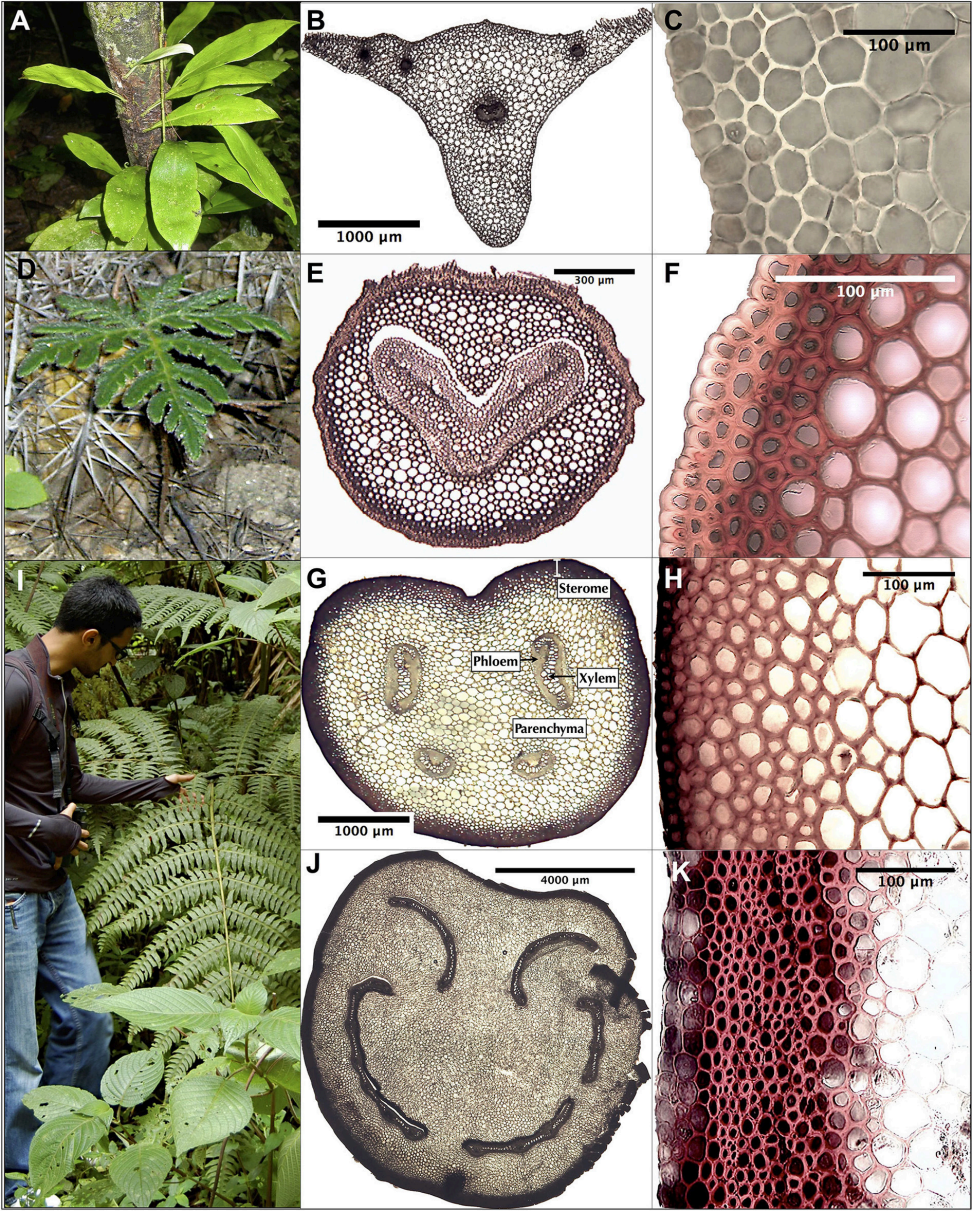
Photos and micrographs of selected Pteridaceae ferns, their petioles and steromes. These species were chosen on account of their diverse habitats, interesting stele arrangements, wide range of leaf sizes and variable steromes, if present. **(A–C)** *Polytaenium citrifolium*, an epiphyte without a sterome; **(D–F)** *Bommeria hispida*, a short, desert-dwelling species with a thin sterome composed of thick-walled fibers; **(G–H)** *Pityrogramma ebenea*, a tropical species with an average sterome but a pronounced adaxial groove in the petiole; **(I–K)** *Pteris livida*, a 2 m tall tropical upland species with relatively large yet poorly reinforced sterome fibers. Informed permission to include student in **(I)** was granted by written consent.

The appearance of the hypodermal sterome in early seed-free vascular plants is a prime example of how modest modification of existing tissues, in this case the progressive lignification of parenchyma, can change the developmental trajectory and functional potential of a plant structure. The sterome is a layer of sclerenchyma fibers that are mostly dead (Dickison, 2000), and present in the majority of ferns just beneath the cuticle of a rhizome or leaf petiole (Figure 1). It has largely been lost in angiosperms (Rowe and Speck, 2004), but some exceptions exist, such as among lianas in young *Aristolochia* and *Manihot* stems that rely on the sterome for self-support prior to their transition to climbing (e.g., Ménard et al., 2009; Wagner et al., 2014). With a Young's Modulus (*E*) that is over a thousand time greater than parenchyma, the sterome can equip plants with up to and over 95% of their axial flexural stiffness (*EI*), leaving the balance to cortical parenchyma and vascular tissue (Rowe and Speck, 2004; Niklas and Spatz, 2012). That early tracheophytes transitioned from small-statured plants with either enations, simple branch systems, or microphylls to plants with potentially huge leaves can be attributed, in no small part, to the appearance of the sterome not only because it stiffened axial organs, but because it created a central, mechanically stable neutral zone in which vascular tissues could be cushioned by surrounding parenchyma (Bateman et al., 1998; Phillips and Galtier, 2005).

The evolutionary transition from parenchyma to sclerenchyma tissue for support may have been relatively simple, requiring little more than the development of a secondary cell wall, cell elongation and enhanced lignification. Fossil evidence of a thick walled sterome characterizing *Cooksonia* axes dates back to the Silurian (Edwards et al., 1986). Similarly, the early Devonian rhyniophytes show some cortical differentiation (Lang, 1917; Kidston and Lang, 1920; Rowe and Speck, 2004) but this is complicated by the arbuscular fungal growth within the cortex. Certainly by the late Devonian, hypodermal steromes were mechanically significant in a range of tracheophytes such as *Psilophyton* and early lignophytes such as *Tetraxylopteris* (Speck and Vogellehner, 1994). Recent phylogenetic analyses place the origin of ferns in the early Devonian (Pryer et al., 2001; Testo and Sundue, 2016), post-dating the earliest occurrences of sterome-like tissue in land plants (Edwards et al., 1986).

The goal of this study was to examine the anatomy, and the geometrical and biomechanical contribution of the hypodermal sterome to leaf support across the highly diverse Pteridaceae family of ferns. The Pteridaceae have origins in the Jurassic, but divergence and expansion are believed to have occurred in the Cretaceous (Schneider et al., 2004; Schuettpelz and Pryer, 2009; Testo and Sundue, 2016) with species venturing into aquatic, brackish, xeric, alpine, understory and epiphytic niches during the Cenozoic (Schuettpelz et al., 2007). Species' life history and morphology is equally diverse; desert adapted cheilanthoids are frequently desiccation tolerant, typically less than 20 cm tall, and active only during the humid seasons (Hevly, 1963; Nobel, 1978), while similarly-sized tropical epiphytes are more or less perennial. Other taxa can be enormous: the cloud forest understory species *Pteris livida* and *P. podophylla* have leaves that exceed 2 m in length, with leaf areas well over a square metre (Figure 1). All of these plants have steromes. Yet equally compelling are members of the Pteridaceae that lack steromes, including the epiphytic vittarioids, and the aquatic ceratopteridoids, that rely on parenchyma and petiole geometry for support and bending resistance.

**Figure 2:**
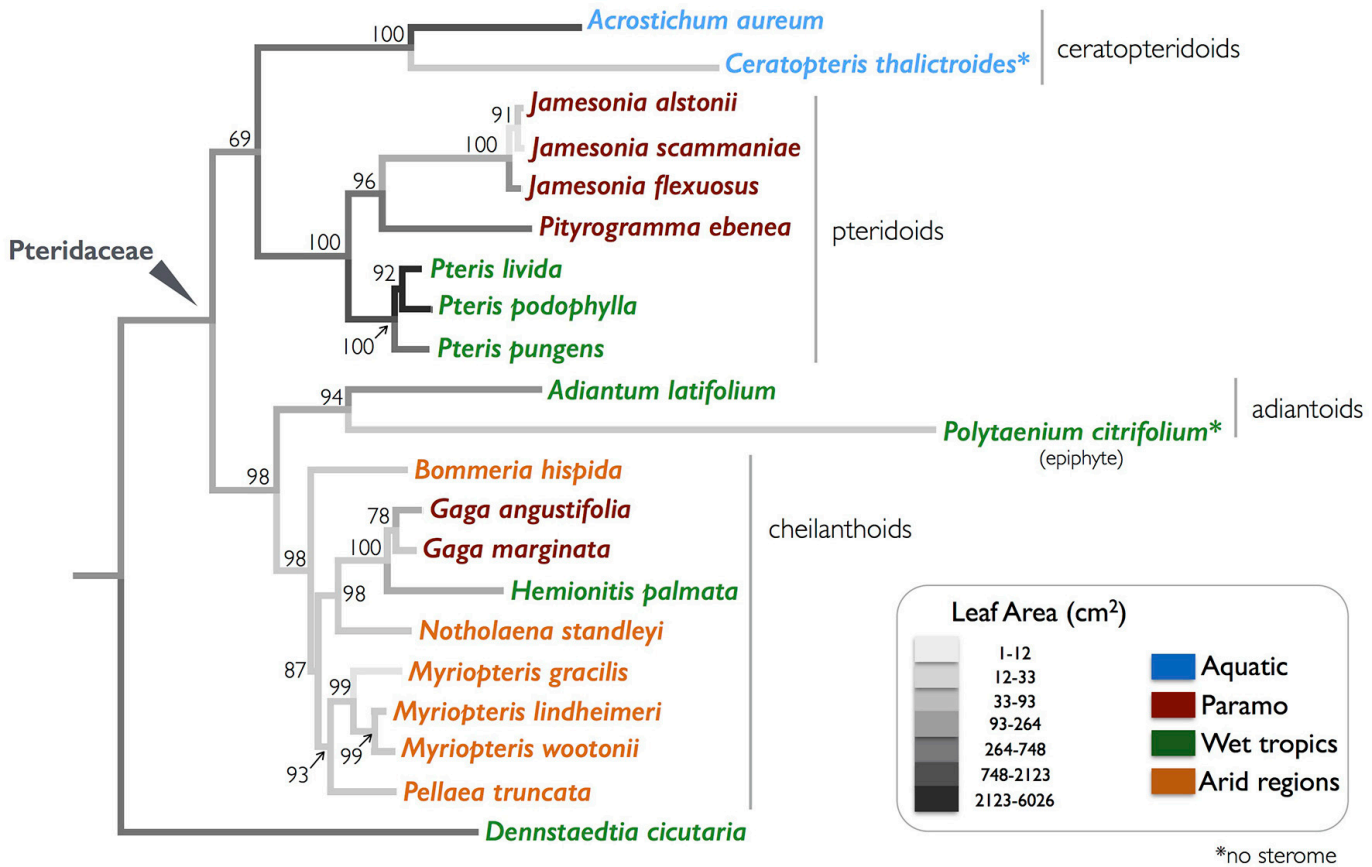
A phylogeny of the selected Pteridaceae ferns examined in this study, with leaf areas mapped onto the branches. The colors of species' name indicate their native habitats. A bootstrap value is associated with each node.

What governs the presence and the radial thickness of the hypodermal sterome in ferns, and how does this trait vary with petiole geometry and leaf size? These questions are explored in the petioles (= stipes) of leaves from 20 members of the Pteridaceae (and one Dennstaedtioid, Figures 1, 2) by first scrutinizing the anatomical attributes of the sterome in terms of cross-sectional area and computing the axial second of moment of area (*I*), and secondly, by looking at the extent to which this geometrical diversity would influence theoretical values of flexural rigidity. The findings challenge the simplest hypothesis that increasingly larger leaves rely on proportionally greater support from the sterome. Rather, it appears that the sterome is a nuanced structure that is related to both support needs and life history traits: in some ferns, its presence may be critical to survival, while in others, it's absence is not a detriment but rather a cost saving.

## Materials and methods

### Plant material

Complete leaves of 20 Pteridaceae species were collected from habitats ranging from aquatic to xeric, and included both terrestrial and epiphytic taxa (Table 1; Figure 2). The tropical, terrestrial *Dennstaedtia cicutaria* was chosen as the outgroup because it belongs to the Dennstaedtiaceae, an earlier-branching sister clade to the Pteridaceae. *D. cicutaria* thus represents the nearest relative outside of the clade of interest (Schuettpelz and Pryer, 2007).

**Table 1:** List of species studied, the clade to which they belong within the Pteridaceae, habitat, and their name abbreviation.

**Species**	**Clade**	**Habit**	**Location/habitat**	**Symbol**
*Acrostichum aureum* L.	Ceratopteroid	Upright terrestrial	Mexico/mangrove forest	Au
*Ceratopteris thalictroides* (L.) Brongn.	Ceratopteroid	Aquatic	Costa Rica/river bank	Ct
*Jamesonia alstonii* A. Tryon	Pteridoid	Upright Terrestrial	Costa Rica/paramo sheltered habitat	Ja
*Jamesonia scammanae* A. Tryon	Pteridoid	Upright Terrestrial	Costa Rica/paramo sheltered	Js
*Jamesonia flexuosus* (Kunth) Copel.	Pteridoid	Scandent Terrestrial	Costa Rica/disturbed cloud forest	Jf
*Pityrogramma ebenea* (L.) Proctor	Pteridoid	Upright-Angled Terrestrial	Costa Rica/disturbed cloud forest	Pe
*Pteris livida* Mett.	Pteridoid	Upright Terrestrial	Costa Rica/cloud forest understory	Pl
*Pteris podophylla* Sw.	Pteridoid	Upright Terrestrial	Costa Rica/cloud forest understory	Po
*Pteris pungens* Willd.	Pteridoid	Upright Terrestrial	Costa Rica/tropical rainforest understory	Pp
*Adiantum latifolium* Lam.	Adiantoid	Upright Terrestrial	Costa Rica/tropical rainforest understory	Al
*Polytaenium citrifolium* (L.) Schuettp.	Adiantoid	Tree-trunk Epiphyte	Costa Rica/tropical rainforest understory	Pc
*Bommeria hispida* (Hook.) J. Sm.	Cheilanthoid	Upright Terrestrial	Arizona/xeric montane habitat	Bh
*Gaga angustifolia* (Kunth) Pryer, Fay W.Li & Windham	Cheilanthoid	Upright Terrestrial	Costa Rica/paramo exposed habitat	Ga
*Gaga marginata* (Kunth) Pryer, Fay W.Li & Windham	Cheilanthoid	Upright Terrestrial	Costa Rica/paramo exposed habitat	Gm
*Hemionitis palmata* L.	Cheilanthoid	Upright Terrestrial	Costa Rica/tropical rainforest understory	Hp
*Notholaena standleyi* Maxon	Cheilanthoid	Upright Terrestrial	Arizona/sandstone canyons	Ns
*Myriopteris gracilis* Fée	Cheilanthoid	Upright Terrestrial	Arizona/sandstone canyons	Mg
*Myriopteris lindheimeri* (Hook.) J. Sm.	Cheilanthoid	Upright Terrestrial	Arizona/xeric montane habitat	Ml
*Myriopteris wootonii* (Maxon) Grusz & Windham	Cheilanthoid	Upright Terrestrial	Arizona/xeric montane habitat	Mw
*Pellaea truncat*a Goodding	Cheilanthoid	Upright Terrestrial	Arizona/xeric montane habitat	Pt
*Dennstaedtia cicutaria* (Sw.) T.Moore	Outgroup: Dennstaedtiaceae	Upright Terrestrial	Costa Rica/tropical rainforest understory	Dc

### The pteridaceae phylogeny

A two-gene (plastid *atpA* and *rbcL*) dataset was assembled in order to resolve relationships among the focal taxa (Figure 2). For each taxon, previously published sequences were obtained from GenBank; accession numbers are provided in Supplementary Data Sheet 1. In five instances, due to the unavailability of suitable sequences from the focal species, it was necessary to use sequences from a closely-related species in the same genus Supplementary Data Sheet 1. The *atpA* and *rbcL* sequences were manually aligned (separately) in AliView version 1.18 (Larsson, 2014). Each of the single-gene alignments was phylogenetically analyzed using a maximum-likelihood approach in RAxML version 8.2.7 (Stamatakis, 2014). These analyses employed the GTRGAMMA model of sequence evolution and involved 1,000 rapid bootstrap inferences followed by a thorough maximum likelihood search. The resulting trees were examined for significant conflicts. Only one such conflict was uncovered among species within the genus *Jamesonia*. The *atpA* and *rbcL* alignments were ultimately combined and analyzed in unison as above, but with parameters independently estimated for each gene. The resulting tree was rooted with the single included representative of the Dennstaedtiaceae, *D. cicutaria*. Schuettpelz et al. (2007) and Prado et al. (2007) present a larger, more complete phylogeny of the Pteridaceae.

### Species collection

Specimens were collected from several localities in Costa Rica, the United States and Mexico. Lowland tropical taxa were sampled at the La Selva Biological Station in Costa Rica (10.4338, −84.0029; elev. 100 m), where annual temperatures range from 19°C to 31°C; this area accumulates over 4 m of annual precipitation. Ferns inhabiting the upland montane cloud forests were gathered from the Parque Nacional Los Quetzales (9.5579, −83.7946; 2,200 m), a region that is generally cooler (18°C to 30°C) and modestly drier (<2 m of rain per year) than the lowland tropics. The Costa Rican páramo systems harbor numerous Pteridaceae, and these were collected at the Cerro de la Muerte (9.5621, −83.7549; above 3,000 m), an alpine community of herbs and shrubs where July temperatures fluctuate from near-freezing at night to over 25°C by mid-day (observed by JP, JEW, and WT).

Leaves of the brackish adapted *Acrostichum aureum* were collected from the shorelines of Lake Sontecomapan (Veracruz, MX; 18.527146, −95.021589), where this species grows profusely. Here, the climate is characterized by year round mild temperatures from 20°C to 30°C, and a relatively dry winter followed by abundant precipitation from June to October.

Dry-adapted Cheilanthoid taxa were sampled from populations in the Mt. Graham region of the Pinolenos Mountain Range in southern Arizona (32.6689, −109.7968; 1,200–1,700 m). The average annual temperature in the nearby town of Safford is 18°C, and mean annual precipitation is 0.25 m, but the sub-montane sites where the plants were collected are cooler and moister than the valley floors. Costa Rican taxa were sampled in June/July of 2014, while the Arizona ferns were gathered during the late August/early September monsoon seasons in 2013 and 2014. All samples were placed in at least two plastic bags with wet paper towels, and transported to the lab within 2–4 days after collection.

### Anatomical measurements and specific leaf area

Cross-sections were made in the mid-petiole region of the leaf. Fern petioles can taper significantly from the leaf base to the rachis mid-point and can show a variety of geometries from the point of insertion through to the tip of the main axis. Since the goal of the study was to employ a comparative measure of petiole geometry that minimized complexities related to the petiole insertion point, the petiole mid-point was retained for the geometrical and mechanical comparisons. This is appropriate for assessing the diversity and functional roles of the sterome in a clear manner; potential problems arising from confounding variation in the petiole properties of leaves from 21 species are thus largely eliminated, revealing patterns that are distinguishable and importantly, readily comparable within the clade.

Anatomical data were collected from *n* = 3 leaves belonging to separate individuals from each species, except in *D. cicutaria* and *P. pungens*, for which only two individuals were available. Mid-stipe cross sections were excised by hand, stained with phloroglucinol to highlight lignified tissues, and mounted in glycerin. The sections were then photographed with a Moticam 2,300 digital camera attached to a Motic BA400 compound microscope (www.motic.com) at magnifications ranging from 20× to 400×.

**Figure 3:**
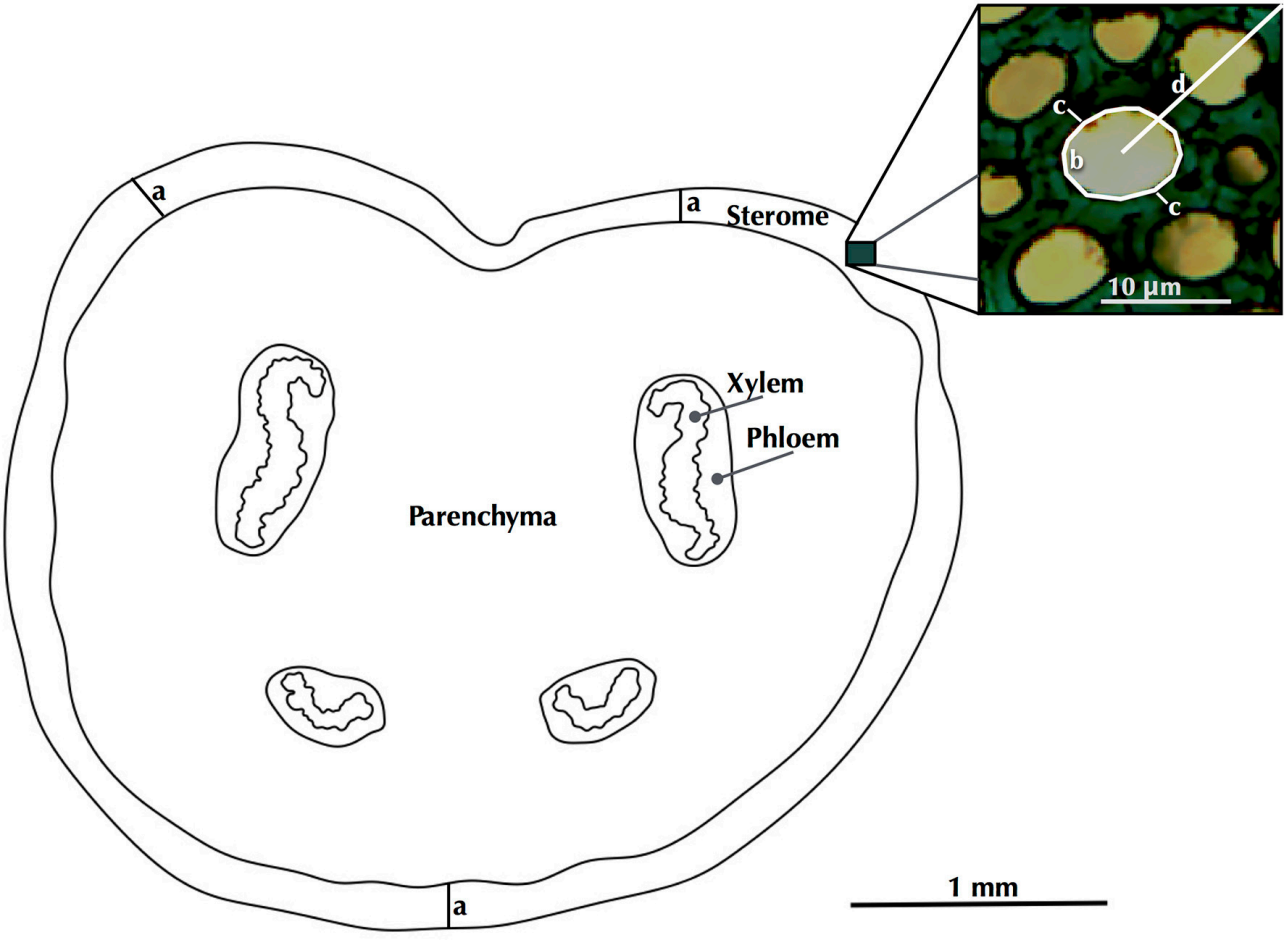
A schematic of a petiole cross section, indicating how sterome thickness (a), fiber lumen area (b), wall thickness (c) and cell distance to cuticle (d) were measured.

ImageJ software (Schindelin et al., 2012) was used to measure morphological and anatomical attributes. No discernible differences were evident in the sterome structure within a cross section, so sterome attributes were measured from three regions that included the adaxial, abaxial and lateral sides. Sterome thickness was defined as the radial distance between the segment cuticle and the innermost extent of the sclerenchyma fibers, just as the tissue transitioned to ground parenchyma (Figures 1, 3). The sterome thickness of each of these sectors was averaged for each leaf, and species' mean sterome thickness was computed as the average of three leaves.

Fiber lumen area, single cell wall thickness and the distance of each cell from the cuticle were measured for each fiber cell within each sector (*N* = 10–44 cells per sector; Figure 3). Lumen area measurements were converted to diameters (*D*) by treating them as area-equivalent circles. The fiber cell wall thickness (*t*) was calculated as the average of two measures, one at the thinnest and another at thickest portions of the cell wall.

The strength of the individual fiber cells in the sterome was estimated by computing the cell wall thickness to lumen diameter ratio (*t/D*). This is a simplified version of a proxy used to estimate the mechanical strength of hollow cells such as xylem conduits (Hacke et al., 2001; Jacobsen et al., 2005; Pittermann et al., 2006). Increasing *t/D* ratios signal stronger, more reinforced cells by virtue of thicker cell walls or narrower lumens.

The fraction of the sterome composed of fiber cell walls (fiber wall fraction; *FWF*) was determined by first subtracting the fiber lumen area from the total fiber area (lumen area + area of one cell wall) for each of the sterome cells, in order to obtain the area occupied by the cell walls. The *FWF* was subsequently computed by dividing the fiber cell wall area by the total fiber area. The fiber wall fraction serves as a proxy for each species' carbon investment in the sterome.

Lastly, leaf length, leaf area and dry mass were determined on the same leaves that were used for the sterome analysis. Leaf area was measured using either a leaf area meter (Li-Cor 3100C; Biosciences, Lincoln, NE) or by scanning the leaves and using ImageJ to analyze the photos. Some leaves suffered handling mishaps, so leaf attributes were measured on digital photos of herbarium specimens collected as close as possible to the sampling sites (Department of Botany Collections, Smithsonian Museum of Natural History, www.botany.si.edu; *Adiantum latifolium, Pityrogramma ebenea, Pteris pungens*, and *Pellaea truncata*; *n* = 3–5 specimens). Photos and field notes guided the selection of specimen size to most closely match taxa observed *in situ*.

Leaf dry mass was obtained by drying the pinnae separate from the stipe and rachis for 48 h at 60°C.

### Tissue cross-sectional area (A), axial second moment of area (I) and influence of geometry on calculated values of rigidity (EI)

A two step approach was used to first measure tissue geometry properties and then explore how different geometries influence theoretical calculated values of *EI* with the assumption that the petiole tissue elements (a) xylem and phloem, (b) cortical parenchyma and (c) sterome (sclerenchyma) were the same between taxa. Different species are unlikely to develop the same Young's modulus for equivalent tissues but it was outside the scope of this study to minutely examine all tissues for all species. The current approach represents a first step prior to more detailed studies in which more tissue-specific mechanical approaches can be used.

Complete petiole cross sections were photographed (see Supplementary Image 1) and outlines of each tissue area were drawn manually (Figure 3). Petiole tissue outlines were orientated in the natural dorsal-ventral orientation, and cross-sectional area and axial second moment of area of each tissue surface was calculated using the commercial image analysis software Optimas (Media Cybernetics, MA, USA), and a specially written macro for calculating axial second moments of area of complex structures (Tancrède Almeras; CNRS, Montpellier, France). The macro establishes the center of mass for the entire cross sectional area and then calculates *I* (mm^4^) of each tissue area with reference to a theoretical neutral line (neutral plane of bending) that passes through the center of mass for the two orthogonal directions relative to the *x* (horizontal) and *y* (vertical) planes of bending. The macro thus computed *I* for all four tissue types and the entire *I* for both the vertical and horizontal directions.

Contributions of each tissue to cross-sectional area and to the axial *I*_total_, a proxy for petiole bending resistance, were computed for both the vertical and horizontal orientations. Like units of cross sectional area (mm^2^) units of second moment of area (mm^4^) are additive, thus the entire axial second moment of area represents the sum of each second moment of area of each tissue (Equation 1)

(1) $$I_{total}=\sum_{i=1}^{m}\;I_{i}$$

where *m* is the number of tissues and *i* is each of the four tissues.

In the second step of this analysis, the theoretical values of rigidity *EI*_theor_ (Equation 2; N m^2^) were computed for both vertical and horizontal orientations for each species using tissue-specific values of Young's modulus according to Niklas (1990), who worked with *Psilotum nudum*. Thus, the following *E*_tissue_ values were applied (MN m^−2^; hydrated tissues): *E*_sclerenchyma_ = 22, 555.3, *E*_xylem_ (primary) = 837.49, and *E*_phloem_ and *E*_parenchyma_ = 18.73.

(2) $$EI_{theor}=\sum_{i=1}^{m}E_{t,i}\;I_{i}$$

where *E*_t_ is the value of Young's modulus (MN m^−2^) attributed to each tissue, *i*.

Tissue properties can differ within a plant axis and certainly among species. For example, the mechanical attributes of branch segments of *P. nudum* can vary over fifty-fold depending on their position along the axis, and more importantly, their age (Niklas, 1990). Among species, *E*_sclerenchyma_ can range from 1,900 MN m^−2^ in dicots to 22,555 MN m^−2^ in *P. nudum* (Niklas, 1990, 1993, 1994). Although much smaller in absolute terms, the *E*_tissue_ of primary xylem and parenchyma is also context or species-dependent (Niklas, 1990, 1993, 1994). It is beyond the scope of the project to identify the species-specific tissue moduli for 21 species of ferns, but given that: (1) sclerenchyma tissue consists purely of mechanically functional and densely packed fibers, (2) all leaves were sampled at full maturity, and (3) *P. nudum* is an early-derived fern (Pryer et al., 2001), it is reasonable to apply Niklas's (1990) *P. nudum* tissue moduli to the taxa examined in this study.

The fractional contribution of each tissue to theoretical flexural rigidity of the petiole, *EI*_contr_ was calculated per Equation (3),

(3) $$EI_{contr}=E_{t,i}\;I_{i}/\sum_{i=1}^{m}E_{t,i}\;I_{i}$$

The integrated, petiole-specific modulus, or theoretical structural Young's modulus, *E*_composite_ (MN m^−2^) is the quotient of *EI/I* (Equation 4), (Speck and Rowe, 1994, 2003). *E*_composite_ is based on attributed values of each tissue type and calculated values of axial second moment of area.

(4) $$E_{composite}=\frac{EI_{theor}}{I_{total}}$$

### Estimates of euler buckling

Solid beams are susceptible to deformation or collapse under excessive loading, a phenomenon known as Euler buckling, which can be successfully modeled in plants (Niklas, 1992). Hence, the final analysis of this study explored the maximum length of the leaf petiole, *L*_max_, that could sustain a given leaf mass (force, *F*, kg m s^−2^) without buckling. This model requires that *EI* be invariable along the length of the petiole due to constant material and geometric properties, but even if some variation exists, the model remains a valuable tool with which to evaluate the comparative mechanical limits of leaf petioles at first pass. Hence, *L*_max_ was computed per Niklas and Spatz (2012):

(5) $$L_{max}={\left(\frac{\pi^{2}EI}{4F}\right)}^{0.5}$$

The safety factor from buckling is *L*_max_/*L*_petiole_, in which *L*_petiole_ is the measured length of the stipe.

### Statistical analyses

Statistical analyses were performed in the R environment (R Core Team, 2015). All data were checked for normality with the Shapiro-Wilks test and log-transformed for analysis, if necessary. The slope and y-intercept (elevation) of the log_x_-log_y_ regression models were computed using the “sma” function in a standardized major axis routine (“smatr” package; Warton et al., 2012). SI units were used for all analyses (Table 2).

**Table 2:** Regression coefficients, scaling exponents and model fits for numerous trait relationships analyzed using standardized reduced major axis regression models *(y* = *ax*^*b*^, where a is the proportionality coefficient related to the elevation (y-intercept) of the fit, and b is the slope on the log-transformed plot; Warton et al., 2012) and phylogenetically independent contrasts (PIC; Paradis et al., 2004).

**Relation**	**$R_{\text{adj}}^{2}$**	***P*-value**	**Slope**	**Elevation**	**PIC: $R_{\text{adj}}^{2}$**	**PIC: *P*-value**
*D_*stipe*_* vs. *L_*frond*_*	0.756	6.34E-07	1.0445	2.12	0.565	5.12E-05
*D_*stipe*_* vs. *A_*leaf*_*	0.883	2.69E-010	2.189	1.193	0.873	3.55E-10
*D_*stipe*_* vs. *M_*leaf*_*	0.704	2.03E-06	2.252	5.604	0.957	1.13E-14
*D_*stipe*_* vs. *T_*sterome*_*	0.822	8.87E-008	0.672	3.889	0.548	7.55E-05
*T_*sterome*_* vs. *L_*frond*_*	0.643	3.60E-05	1.534	−3.856	0.2838	0.0076
*T_*sterome*_* vs. *A_*leaf*_*	0.77	8.02E-07	3.233	−8.804	0.3	0.0061
*T_*sterome*_* vs. *M_*leaf*_*	0.613	7.40E-05	3.243	−7.115	0.447	5.48E-04
*T_*sterome*_* vs. *T_*wall*_/D_*lumen*_*	0.794	3.09E-007	−0.952	5.944	ns	ns
*T_*sterome*_* vs. *D_*lumen*_*	0.706	6.67E-006	0.683	−0.493	0.701	1.34E-06
*T_*sterome*_* vs. *FWF*	0.63	5.05E-05	−4.29	1.49	ns	ns
*Distance* vs. *T_*wall*_/D_*lumen*_*	0.44	2.20E-16	−0.627	1.96	–	–
*%_*sclerenchyma*_*vs. *%_*I*_*	0.998	2.22E-16	0.894	0.383	0.99	2.20E-16
*%_*parenchyma*_*vs. *%_*I*_*	0.659	1.41E-05	1.383	−0.747	0.526	1.79E-04
*EI* vs. *A_*leaf*_*	0.77	8.02E-07	3.233	−8.804	0.241	0.0163
*I* vs. *A_*leaf*_*	0.858	4.68E-09	0.583	−2.077	0.307	6.60E-03
*L_*petiole*_* vs. *EI*	0.824	7.99E-08	3.505	0.012	0.1369	0.0587
*F* vs. *EI*	0.738	1.24E-06	1.844	2.103	0.265	0.012

*D_stipe_, stipe diameter (m); L_frond_, frond length (m); L_petiole_, petiole length (m); A_leaf_, leaf area (m^2^); M_leaf_, leaf dry mass (g); T_sterome_, sterome thickness (μm); T_wall_/D_lumen_, fibre single cell wall thickness/fibre lumen diameter; FWF, fibre wall fraction, %_sclerenchyma_ and %_parenchyma_, the percentage of the cross-sectional petiole area composed of sclerenchyma and parenchyma respectively; %_I_, the percentage contributed by either sclerenchyma or parenchyma to I; EI, flexural rigidity, (Nm^2^); I, second moment of area (mm^4^); F, force (N)*.

A comparative approach to studying the function and evolution of continuous traits acknowledges that taxa are descendants of a common ancestor, and that taxon relatedness violates the key statistical assumption that data are independent (Felsenstein, 1985; Garland et al., 1999). Phylogenetic independent contrasts (PICs) compensate for non-independence by accounting for tree topology and branch length in calculations of phenotypic differences between related taxa (Felsenstein, 1985; Garland et al., 1999). Hence, PIC analyses were performed with the “ape” package in R using the Pteridaceae topology (Figure 2; Table 2; Paradis et al., 2004). The tree was pruned using the “drop.tip” function as necessary to account for missing *I* and *EI* data for *Acrostichum aureum*. Petioles in this species were too large and brittle to generate a stitched, composite photograph of the entire petiole cross-section with any confidence. Lastly, the simple leaf area reconstruction in Figure 2 was generated using the ‘Parsimony Ancestral States’ function in Mesquite 3.04 (Maddison and Maddison, 2015).

## Results

### Leaf size and morphology

The phylogeny in Figure 2 illustrates the four major clades in the Pteridaceae, highlighting the wide range of leaf sizes among the focal taxa as well as their habitat diversity. Leaf area varied by over three orders of magnitude from 5.58 ± 3.59 cm^2^ (mean ± SD) in the desert-dwelling *Myriopteris gracilis* to 5,968 ± 2,649 cm^2^ in *Pteris livida*, which occupies the wet tropical understory. With the exception of the tropical *Hemionitis palmata*, which had a leaf area of 50.25 ± 16.46 cm^2^, the New World cheilanthoids examined here were were diminutive, with leaf areas well below 40 cm^2^. Species that occupy the paramo—a high-elevation habitat in which plants are exposed to extreme temperature changes, high winds and high levels of solar radiation—are also small and grow close to the ground such as *Gaga* spp., or nestle in the vegetation under the elfin trees and shrubs like *Jamesonia* spp. There was no clear pattern with respect to leaf type: cheilanthoid taxa were just as likely to have highly divided leaves (bipinnate pinnatifid) as the large *Pteris* species.

**Figure 4:**
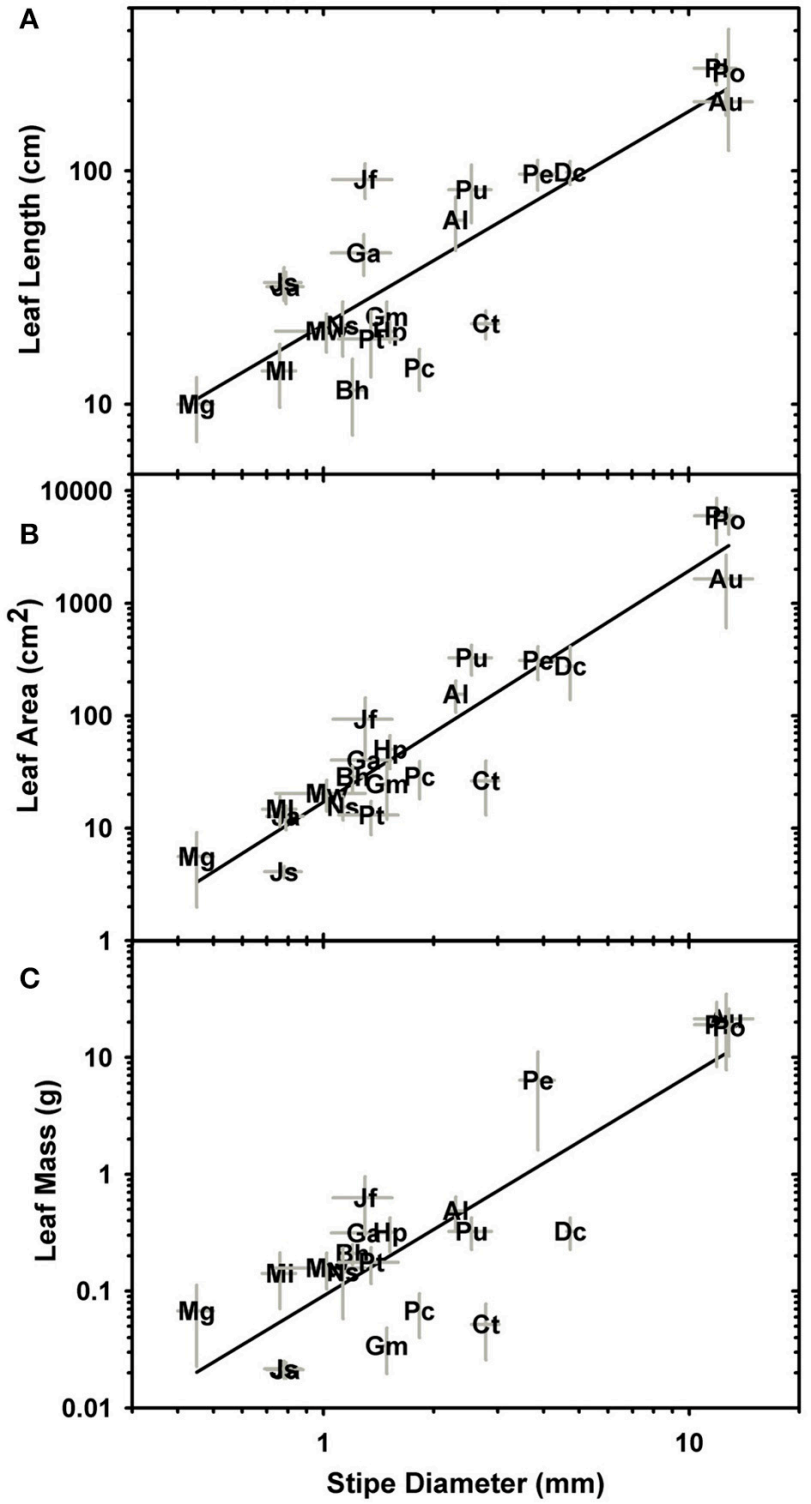
Log-log plots of stipe diameter in relation to leaf length **(A)**, leaf area **(B)**, and leaf mass **(C)**. Table 2 provides the scaling and correlation coefficients.

**Figure 5:**
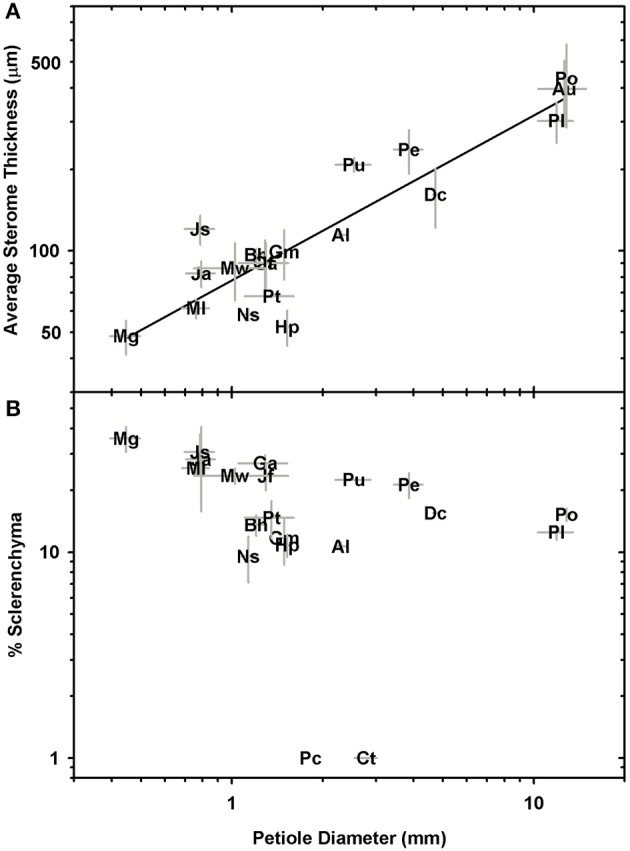
Log-log plots of petiole diameter vs. sterome thickness **(A)** and the percentage of sclerenchyma per petiole cross-section (**B**; no relationship).

Significant linear correlations were observed between petiole diameter and leaf length, leaf area and dry mass with *R*^2^ values of 0.76, 0.88, and 0.7, respectively (Figure 4; Table 2; Supplementary Data Sheet 2). The results from phylogenetic independent contrasts analyses (PICs) corroborate these findings (Table 2). A similarly close and significant association was observed between petiole diameter and the thickness of the sterome, with stipe diameter explaining over 82% of the variation in sterome thickness (Figure 5A; Table 2). No relationship was observed between petiole diameter and the % of the petiole occupied by the sterome—the sterome fraction is constant across leaf size in the Pteridaceae. Taken together, the data indicate that leaf biomass scales predictably with petiolar dimensions and sterome thickness; this accords with the commonsense expectation that the sterome of the petioles serves to mechanically support the leaf laminae.

### Sterome organization and fiber cell anatomy

**Figure 6:**
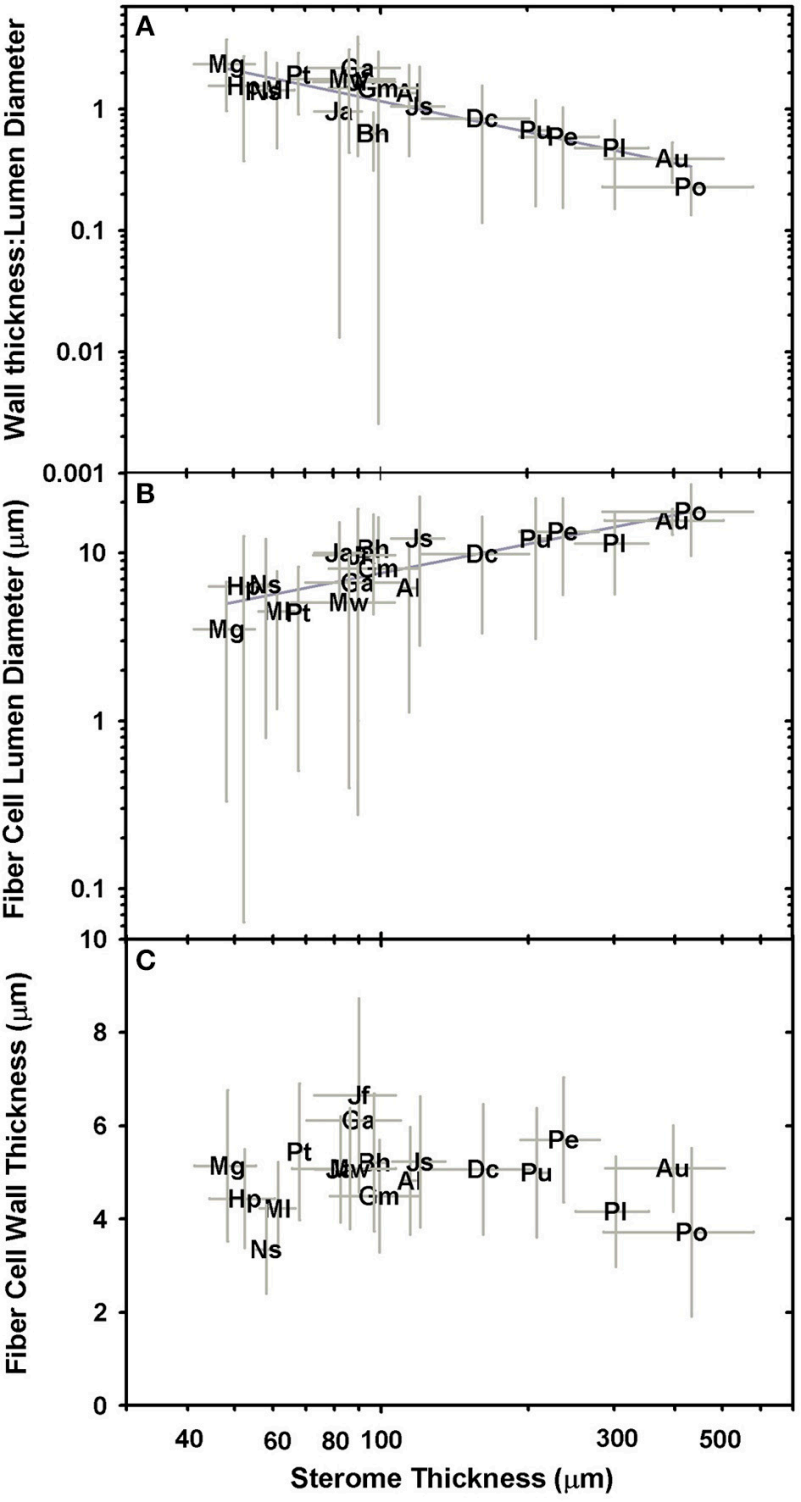
Log-log plots of relationships between sterome thickness and wall thickness:lumen diameter (**A**; *t/D*), sterome cell lumen diameter **(B)**, and sterome wall thickness **(C)**.

**Figure 7:**
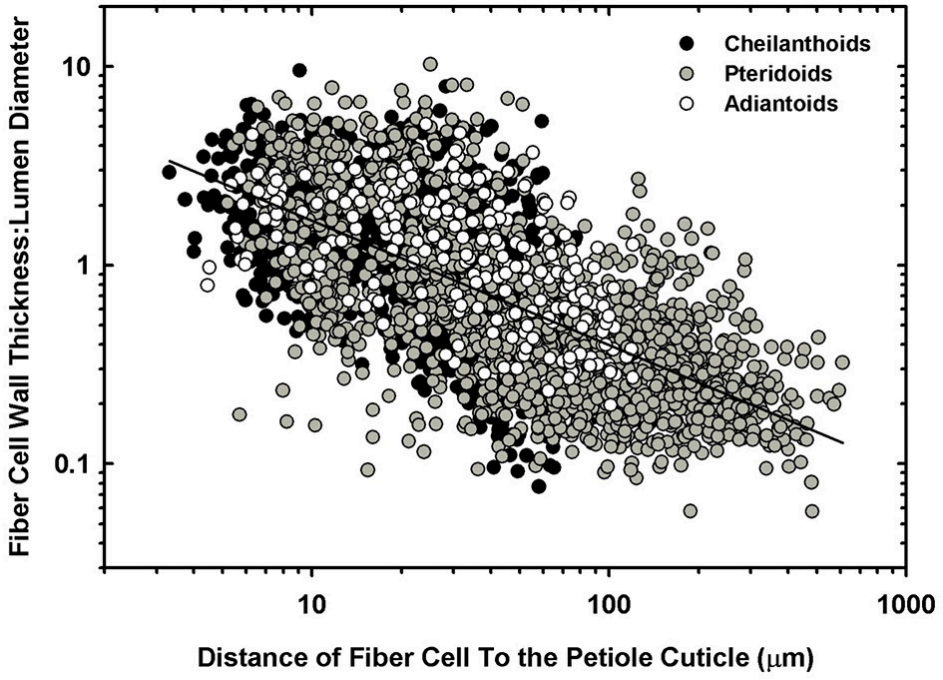
A log-log plot of the distance of individual sterome fibers from the petiole cuticle in relation to their degree of reinforcement (*t/D*).

The cellular composition of the sterome may determine in part its mechanical properties, so the lumen diameter and single wall thickness of the fiber cells were measured, as was the cells' distance from the cuticle. The *t/D* ratio decreases with progressively thicker steromes, which suggests that the cost of thicker steromes, such as in *Pteris* spp. and *Acrostichum* may be partially offset by “cheaper,” less reinforced cells (Figure 6A). By contrast, the thin steromes found in the cheilanthoid clade, and in several pteridoids, are composed of cells with higher *t/D* ratios. Unlike fiber wall thickness, which remains invariable across the range of sterome widths, it is lumen diameter that determines the *t/D* of fiber cells, ranging from 3.52 ± 3.19 μm in *Myriopteris gracilis* to 17.61 ± 7.94 μm in *Pteris podophylla* (Figures 6B,C). Interestingly, cells with the highest *t/D* ratios are located closest to the periphery of the stem, just underneath the cuticle where tension and compression exert the highest degree of stress during bending (Figure 7; Niklas, 1992). This pattern was evident across the three major Pteridaceae clades.

**Figure 8:**
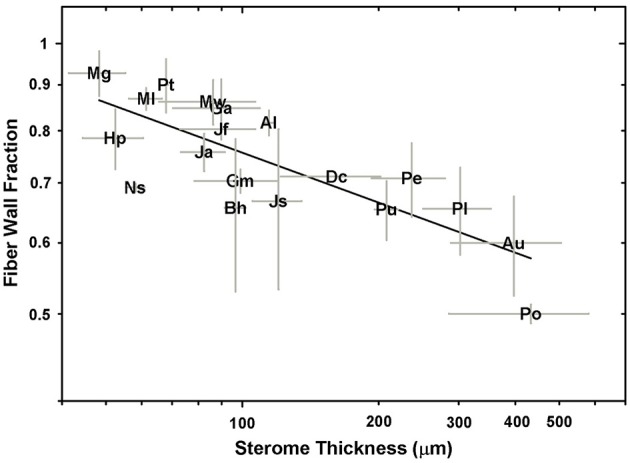
A log-log plot of sterome thickness vs. the fiber wall fraction of the sterome.

The data in Figures 6, 7 indicate that species with narrow steromes are composed of more reinforced fibers, in which a high *t/D* ratio is achieved in part by reduced lumen area. This implies that the relative carbon investment in sterome composition increases in small-statured species and this is indeed the case. Ferns with narrow stipes develop denser steromes on account of a higher fiber wall fraction, relative to larger-leaved taxa (Figure 8). For example, with an average stipe diameter of 0.45 ± 0.05 mm, *M. gracilis* is the smallest fern sampled in this study, yet the fiber wall fraction of its sterome is 0.93 ± 0.05. By contrast, only 50% of the sterome is occupied by fiber walls in *P. podophylla*.

### Estimates of second moment of area and flexural rigidity

**Figure 9:**
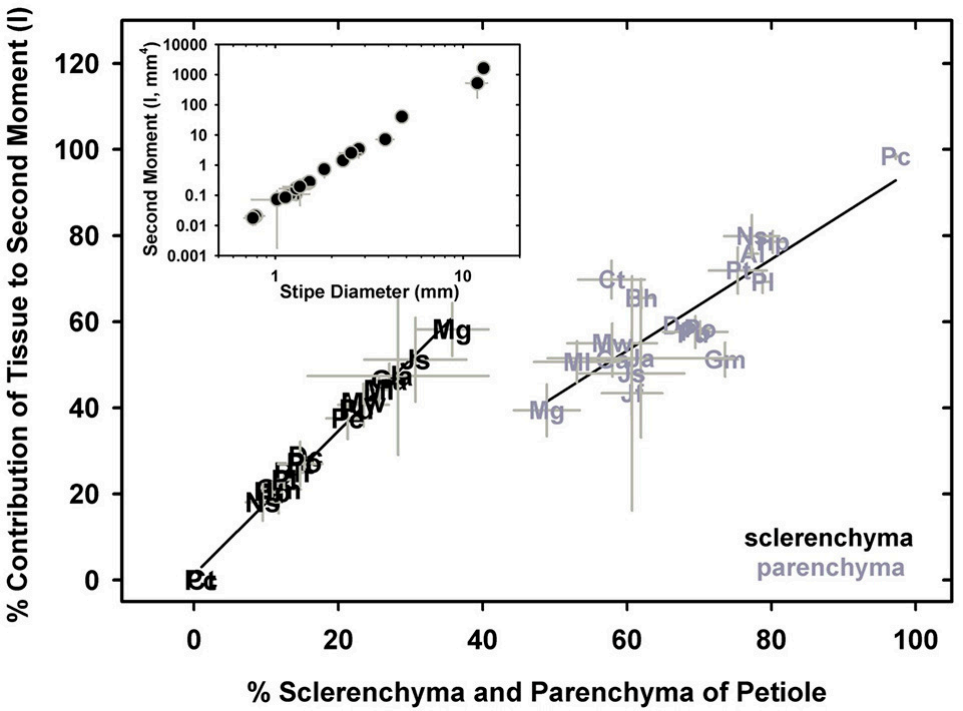
The percentage of sclerenchyma and parenchyma tissue occupying a cross-section of species' petioles in relation to each tissue's contribution to the second moment of area. The inset shows the relationship between petiole diameter and the second moment of area.

Measurements of petiole axial second moment (*I*) varied by six orders of magnitude, from 0.002 ± 0.001 mm^4^ in *M.gracilis*, to 1,628 ± 108 mm^4^ in *P. podophylla*, which had stipe diameters of 0.45 ± 0.05 mm and 12.84 ± 0.44 mm, respectively (Figure 9, inset). Parsing the *I* by tissue type revealed that it is the sclerenchyma and parenchyma that contribute most to the overall stipe *I* by virtue of their position in the petiole (Figure 9), with xylem and phloem responsible for less than 15% of overall *I* (data not shown). Sclerenchyma occupies the highest percentage of the petiole and contributes most to *I* in small species such as *M. gracilis* and *Jamesonia scammaniae*, whereas as large taxa such as *P. podophylla, P. livida* and *Dennstaedtia cicutaria* allocate less than 20% of cross-sectional petiole area to sclerenchyma, relying instead on bulky geometry to achieve high *I*. The second moment is solely a function of petiole geometry (calculations of *I* ignore material properties) so the sterome itself is not the sole anatomical trait necessary to achieve high second moment of area. However, its peripheral position, combined with its high *E*_tissue_ enhances its mechanical contributions. This combination of geometry and mechanical strength presents a functional advantage with respect to petiole flexural rigidity, *EI*.

**Figure 10:**
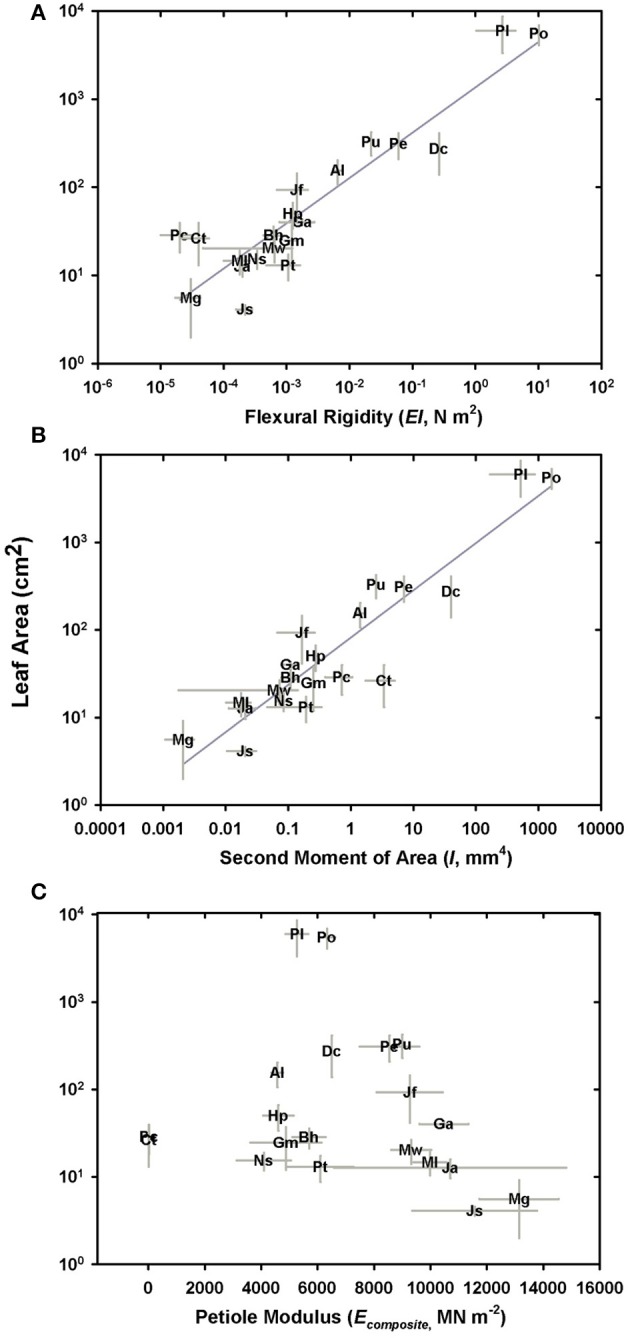
Components of petiole mechanical properties in relation to species leaf area. **(A)** Log-log plot of flexural rigidity vs. leaf area; **(B)** Log-log plot of the second moment of area in relation to species leaf area; **(C)** The petiole composite modulus of elasticity vs. leaf area (no relationship).

While estimated flexural rigidity (*EI*_theor_) reflects the additive contributions of the four tissues that comprise the petiole, the data indicate that it is the second moment of area in combination with the material properties of sclerenchyma (high modulus of elasticity) that drives variation in flexural rigidity (Figures 10A,B). The overall modulus of the petiole (*E*) plays no role (Figure 10C). The moment and flexural rigidity are in turn, closely related to leaf area. Separately computing flexural rigidity for each of the four tissues considered both the tissue modulus and its second moment of area, revealing that the sterome contributes over 97% to the total petiole flexural rigidity across all species. From a practical standpoint, this means that sterome was responsible for 98% of the flexural rigidity in the stipe, in both *M. gracilis* and *P. podophylla*, despite the vast difference in species petiole stiffness. This reflects the essential role of the high Young's modulus and peripheral position of the sclerenchyma tissue. No clear overall relationship was observed between the petiole modulus and leaf area (Figure 10C), a result that most likely reflects the structural heterogeneity of the petiole (Figure 4B). Lastly, the average ratio of the estimated flexural rigidity in the *x* and *y* axial directions (*EI*_x_*/E*_y_) is 0.88, meaning that the petioles are stiffer along the dorso-ventral axis, rather than in a lateral direction. This is consistent with a more often greater vertical diameter of petioles, often combined with a dorsal notch in the petioles of most species, and reflects selection for reinforcement in response to top-loading (falling debris, rain) rather than lateral disturbance.

**Figure 11:**
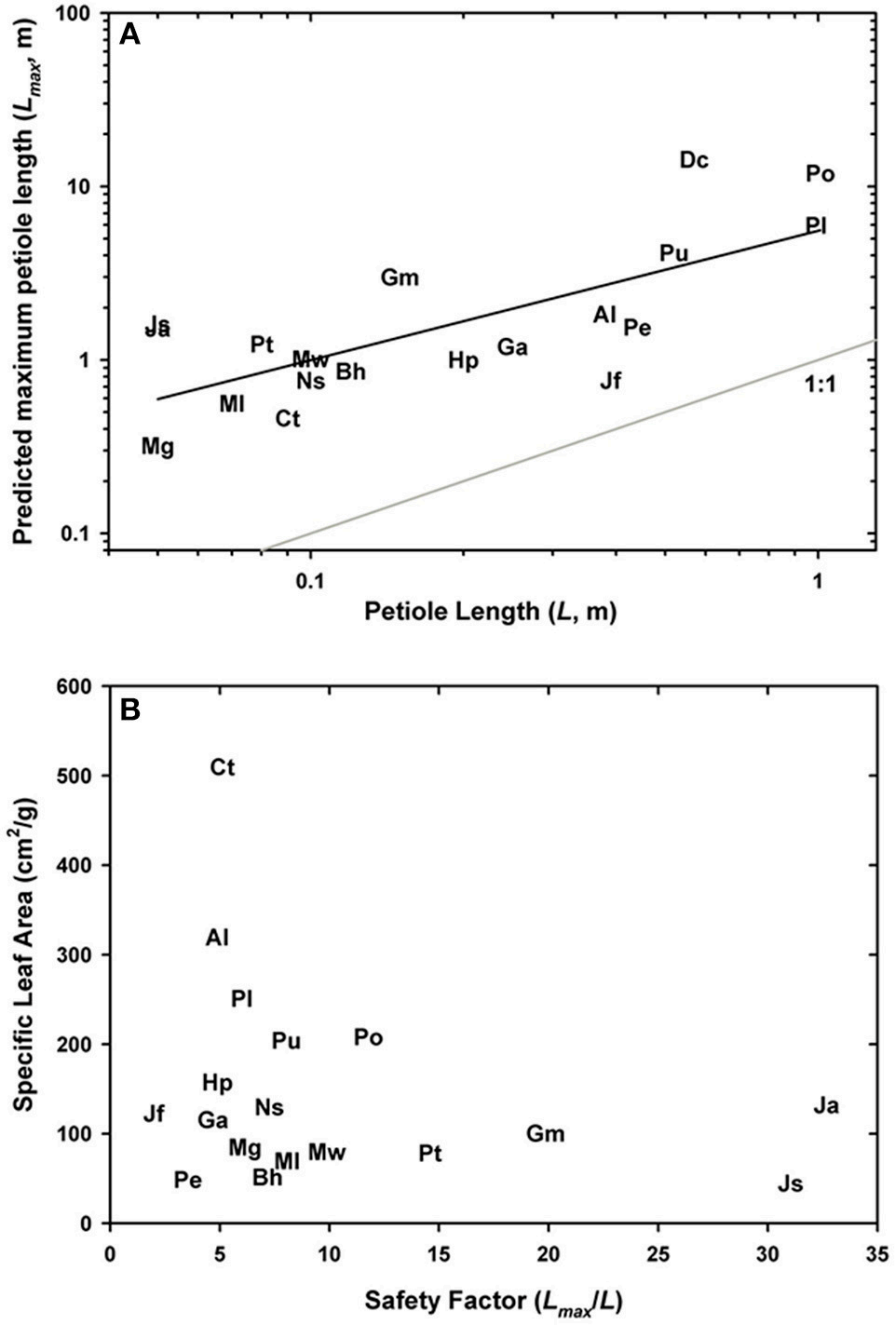
Log-log plot of measured petiole length in relation to the predicted maximum petiole length **(A)**. The relationship between the safety factor from buckling and leaf investment, that is specific leaf area, is shown in **(B)**.

The final analysis explored the relationship between *L*_max_, the maximum sustainable petiole length and leaf investment. Figure 11A shows that *L*_max_ was consistently longer than the measured petiole length, with the safety factor averaging 11.2 ± 2.19 across all taxa surveyed. Given that dry mass of the leaves was used without the weight of the petiole (a lighter load would artificially increase *L*_max_), that *L*_max_ was computed from *EI* values calculated from mid-stipe rather than the slightly thicker basal end of the leaf (this may decrease *L*_max_ by underestimating *EI*), and that tissue moduli may vary between species, the resulting values of *L*_max_ and the associated safety factors may deviate from absolute measures. Yet despite these caveats, meaningful patterns emerged. For example, the highest safety factors were not observed in the largest ferns such as *P. podophylla* and *P. livida* as expected, (safety factors were 11.8 and 8, respectively), but rather in the *Jamesonia* species which had safety factors in excess of 30. Such high safety factors result from a combination of high *EI* and to some extent, low leaf area, both common attributes in these páramo ferns. In fact, the safety factors in many of the small ferns sampled for this study may be significantly higher than reported because their stipes contain lignified parenchyma, a distinct tissue from sclerenchyma characterized by large cells with thicker walls that stain with phloroglucinol (Supplementary Image 1). Lignified parenchyma was observed in the cheilanthoids, as well as a subset of pteridoids, and may increase the petiole *EI* of these plants.

Lastly, the relationship between *L*_max_/*L*_petiole_ and specific leaf area indicates an important pattern: ferns are unlikely to invest in strong, deformation-resistant petioles unless they are coupled with similarly robust (low SLA) leaves (Figure 11B). By contrast, species with low flexural rigidity such as the aquatic *C. thalictroides* will have thin, delicate leaves with a high specific leaf area, a strategy consistent with the turbulent habitat and rapid life cycle of this species. Taken together, these results signal an interesting co-ordination between leaf biomechanics and life history strategy.

### Discussion

The main goals of this research were to examine the structural attributes of the hypodermal sterome across ferns from diverse habitats, and to identify how these traits relate to leaf size and mechanical needs. The sterome contributes over 97% to overall petiole flexural rigidity, but in contrast to initial expectations, the sterome fraction does not increase with leaf size. Rather, allometric analyses revealed consistent proportions between stipe diameter, sterome thickness and leaf area (Figures 4, 5; Table 2). Taken together, the results indicate that the sterome is under strong selection for support of the leaf. In the absence of a sterome, fern petioles can best be described as parenchymatous hydrostats that are either cheap and flexible (lower *EI*_theor_) as in the case of the fast-growing rheophyte *C. thalictroides*, or highly reduced and buttressed, as in the epiphytic *P. citrifolium*, which has relatively robust but small leaves (Figure 1A). On the other hand, sterome-bearing petioles exploit a combination of geometrical traits (*I*) coupled with high tissue modulus (*E*_sclerenchyma_) to develop strong petioles that are resistant to buckling (Figures 9, 10). Consistent with this is the co-ordination between petiole safety factor and specific leaf area: species with strong stipes will invest in durable leaves with low SLA, while the combination of tough petioles, and thin leaves with high SLA appears to be functionally incompatible (Figure 11). Taken together, this study reveals meaningful patterns in biomechanics that align with species' leaf size, sterome attributes and life-history strategy.

Results of this study support the idea that the presence of the sterome released ferns from a number of physiological and mechanical constraints to explore a diversity of habitats and leaf morphologies. This was examined by Niklas (1991), who considered the possibility that water deprivation may select for hypodermal thickening to compensate for turgor loss. Certainly many cheilanthoid ferns, including *Notholaena standleyi, B. hispida* and the *Myriopteris* species tolerate desiccation during the dry season (Hevly, 1963; Rothfels et al., 2008), and thus rely on the sterome for support during this dormant phase. Tropical ferns such as *Pteris* and *Dennstaedtia* exploit parenchymatous hydrostats in combination with external sclerenchyma to achieve significant gains in rigidity, while at the very opposite end of the spectrum, riparian habitats selected for the loss of the sterome in *C. thalictroides*. Selection for a mechanical sterome for certain life forms and habitats has played out numerous times during diversifications of the major clades. Rowe and Speck (2004) suggested that during the diversification of early land plants, the appearance of the sterome presented a significant structural leap that allowed certain early-derived growth forms to shift away from hydrostatic support, and evolve in both size and complexity. Certainly the largest ferns in this study all possess a sterome. Most tellingly, both *A. aureum* and *C. thalictroides* occupy riparian-brackish habitats, but the sterome in *A. aureum* supports leaves that are nearly 8× longer than those of its sterome-free counterpart (Figure 4). Of course, each species has a unique life history in which reproduction, habitat and length of the growing season are factored into cost-benefit trade-offs, so the evolution of the sterome may be nuanced because this structure represents a substantial carbon investment (Figure 8), that may or may not be adaptive under a given set of circumstances.

The strategic placement of the sterome near to the periphery of the petiole allows ferns to optimize the bending resistance conferred by second moment of area combined with the inherent stiffness of sclerenchyma fibers. Functionally, this means that the petiole is well-protected from the tensile and compressive stresses that increase with distance from the center of the petiole. By contrast, the centrally placed vascular tissues play almost no role in mechanical support, despite the relatively high modulus of xylem tissue. It is this decoupling of support and transport that allows fern tracheids to be so much wider and longer than those of woody plants, rivaling the hydraulic efficiency of both conifer and angiosperm xylem (Pittermann et al., 2006, 2011, 2015). Woody angiosperms employ a markedly different strategy, in which leaves are tethered to woody stems by flexible petioles, whereas some herbaceous taxa rely on petioles with a combination of peripheral bundles of sclerenchyma, collenchyma, as well as turgor to elevate their leaves. Fern petioles exist in the morphospace between woody stems and angiosperm petioles: unable to produce secondary tissues and (for the most part) vessels, the sterome provides a relatively simple structural solution that separates stiff axial support from efficient hydraulic function to achieve a high degree of leaf functional flexibility.

The deep-time ancestry and near-ubiquitous presence of the sterome in ferns suggests that this tissue should be a target of selection. Ideally, further mechanical tests on specific tissues would provide insight into the empirical rather than modeled responses of the sterome, but even with this limitation, this study provides perspective on important structure-function relationships of this tissue. For example, the absolute thickness of the sterome varies proportionally with stipe diameter and leaf size, but the fraction of the stipe that is occupied by sclerenchyma is both unrelated to stipe diameter and has no overall bearing on the flexural rigidity of the stipe (Figure 5; Supplementary Image 2). Contrary to expectation, sclerenchyma comprised the largest proportion of transverse petiole area in the *smallest* ferns, such as the cheilanthoids and the *Jamesonia* species. Despite their large sterome fraction, these plants had significantly lower estimated flexural rigidity than larger ferns, which capitalized on second moment of area to achieve bending resistance (Figure 10). Relying on *I* for deformation resistance implies that large species can invest less in their sterome, as indicated by fiber cells with low *t/D* ratios (Figure 7) and lower fiber wall fraction (Figure 8). In the small Pteridaceae ferns, relatively thin steromes require stronger fibers to presumably compensate for the low second moment of area of their narrow stipes. Cheilanthoids and *Jamesonias* probably need a tough sterome: growing in deserts and paramos, they are subject to wind and extreme climatic conditions, so strong leaves are necessary to ensure carbon gain during relatively narrow windows of opportunity.

The transition from parenchymal hydrostats to fibrous steromes occurred early in the evolution of tracheophytes. Early land plants such as *Aglaophyton major* exhibit parenchymal differentiation that Rowe and Speck (2004) interpreted as sterome-like, though it might also be linked with arbuscular fungal interactions (Taylor et al., 2004). However, the stems of the Devonian *Psilophyton dawsonii* and the lignophyte *Tetraxylopteris schmidtii* do present well-developed, though differently organized mechanical steromes. Thus, mechanically efficient hypoderms with sclerenchyma or sclerenchyma-like tissue appear more frequently in derived Devonian tracheophytes (Rowe and Speck, 2004). The presence of lignified cortex tissue in the cheilanthoid ferns, as well as the two *Jamesonia*s suggests that a similar transition from simple to reinforced parenchyma may have occurred in the Pteridaceae. In these species, transverse petiole sections revealed parenchyma cells with lignified walls (Figures 1C,D, *B. hispida*; Supplementary Image 1). Lignified parenchyma is present in some monocots and dicots, and Niklas (1991) observed it in *P. nudum*, but whether lignin is located in primary or secondary cell walls is unclear (Grosser and Liese, 1971; Dietz and Ullmann, 1997; Evert, 2006). In ferns, fortified parenchyma may structurally enhance narrow petioles and thus compensate for their low values of *I* because this tissue probably has a higher elastic modulus than typical parenchyma (see Niklas, 1991). Hence, current estimates of *EI* in some of the focal Pteridaceae may be lower than they actually are. In addition to analyzing experimentally the mechanical properties of different sterome tissues and petioles, future work should examine the development, phylogenetic distribution and functional significance of lignified parenchyma; it is possible that interesting ground tissue adaptations to drought may have been long overlooked.

The presence of the sterome has a “strategic” impact on petiole *EI* due to the high elastic modulus of sclerenchyma tissue, as well the peripheral placement within the petiole. By comparison, the parenchyma and the vascular tissues contribute less than 3% to *EI* largely due to their low elastic moduli (Niklas, 1999b). Among the surveyed Pteridaceae species, *I* varied from 0.004 mm^4^ in *M. gracilis* to 1,628 mm^4^ in *P. podophylla*, consistent with earlier data reported for angiosperm and fern leaves (Niklas, 1991; Ennos et al., 2000; Masselter et al., 2007), and unlike the petiole composite modulus (*E*_composite_), *I* exerted a strong effect on petiole *EI* (Figure 10). These patterns have been reported in previous studies of leaf and stem biomechanics (Niklas, 1991; Rowe et al., 1993; Speck and Rowe, 1994, 2003). In the Pteridaceae, estimated flexural rigidity varied from 2.7 × 10^−5^ in *M. gracilis* to 10.15 N m^2^ in *P. podophylla*, averaging 0.66 ± 2.31 N m^2^, so quite similar to the 5.8 × 10^−4^ to 0.844 N m^2^ range reported for four fern species by Niklas (1991), and well within the 0–100 N m^2^ range of measures obtained by mechanical testing of angiosperm and fern petioles (Niklas, 1991, 1999b; Vogel, 1992; Ennos et al., 2000). Modeling *EI*_theor_ is inherently imperfect but nevertheless, the comparative approach identifies the means by which highly diverse geometric organizations potentially influence the mechanical properties of the fern petiole. Indeed, comparable models have been successfully applied to fossil stem material, revealing how *EI* varies with ontogeny even in taxa with no modern analogs (Rowe et al., 1993; Speck and Rowe, 1994, 2003; Masselter et al., 2007).

The structure of fern leaves is generally more canalized than that of angiosperm leaves, but the Pteridaceae show exceptional diversity in form and function. Dicot leaf petioles typically do not have a sterome, and instead rely on collenchyma and parenchyma for flexible support in combination with a multitude of petiole geometries (Vogel, 1992, 2012; Ennos et al., 2000). *Polytaenium* and *Ceratopteris* have some collenchyma below the dermal cells, but by and large, most Pteridaceae ferns occupy relatively calm understory or sub-canopy habitats where rain and falling debris impose the most mechanical stress; here axial rigidity is perhaps more important than torsional bending (Niklas, 1999a,b). That said, numerous variables may explain why some ferns have such overbuilt petioles, which is manifest in the high safety factors from buckling. For example, some species must contend with a high-disturbance understory, while others must maintain their structure in spite of recurring episodes of dehydration and rehydration. However, among a number of possible reasons for overbuilt rigidity and a robust sterome is a compelling life history trait, that is the presence of spores on the underside of select leaves. There is little point in producing feeble fertile fronds. Among the Pteridaceae, the L_max_/L_petiole_ averaged to 11.2 ± 2.19 (±SD), similar to the 9.73 ± 1.53 (±SE) reported by Niklas (1994) for ferns. Safety factors in dicot herbs, palms and trees are less than 8.22, 2.78, and 4.66 by comparison (Niklas, 1994). The co-ordination between investment in petiole structure (safety factor) and specific leaf area suggests that like angiosperm leaves, fern leaves are subject to predictable economic trade-offs arising from their habitat, life-history strategy and quite possibly, their reproductive attributes (Wright et al., 2004; Karst and Lechowicz, 2007).

The strong associations among leaf traits in this study are consistent with earlier work in both ferns and dicots (Niklas, 1999a,b; Limm and Dawson, 2010; Watkins et al., 2010; Pittermann et al., 2011, 2015; Burns et al., 2016). Importantly, the mechanical scaling exponents for relationships between petiole length, *EI* and laminar load (*F*) vs. *EI* (3.505 and 1.844, respectively, Table 2) align with those of Niklas (1999b), whose work on pinnate and palmate leaves produced exponents of 2.45–3.24 for *L*_petiole_ vs. *EI*, and 1.63–2.34 for *F* vs. *EI*. The tight scaling between elements of leaf morphology and leaf mechanics points to an overall co-ordination of leaf structure, and physiological and mechanical function, the basis of which has been attributed to resource distribution (West et al., 1999; Savage et al., 2010). With only primary xylem and limited leaf venation, reduced hydraulic capacity may constrain fern leaf size and physiology more directly than in angiosperms, which benefit from the hydraulic flexibility conferred by high vein density (Brodribb et al., 2007; Katifori et al., 2010; Pittermann et al., 2011; Scoffoni et al., 2011).

The Pteridaceae family of ferns is exceptionally diverse and thus an ideal system for investigating evolutionary ecophysiology of leaf form and function. The results of this study reveal that despite expansion into a broad range of habitats, leaf and sterome attributes converge upon predictable, isometric relationships; however, habitat and life history must inevitably inform any discussion regarding the mechanical properties of steromes and petioles. Future studies will extend these findings by integrating species' water and carbon relations with leaf biomechanics.

## Author contributions

JM co-designed the study and collected data; NR provided critical analytical tools, insights and comments on the manuscript; AB collected data; ES built the Pteridaceae phylogeny; JEW, JKW, KM, MW, WT, and JB collected plant material in Costa Rica, Mexico and Arizona, and provided valuable guidance; JP co-designed the study, collected plant material, collected data, performed analyses and wrote the manuscript with input from NR.

### Conflict of interest statement

The authors declare that the research was conducted in the absence of any commercial or financial relationships that could be construed as a potential conflict of interest.

## Supplementary material

Supplementary Image 1 Additional petiolar cross-sections from the surveyed Pteridaceae taxa.

Supplementary Image 2 Non-significant relationships between the percentage of sclerenchyma that occupies the petiole and flexural rigidity **(A)** and leaf area **(B)**.

Supplementary Data Sheet 1 A list of Pteridaceae species, Genbank *atpA* and *rbcL* sequence ID numbers, and source attributions.

Supplementary Data Sheet 2 A pair-wise correlation matrix of the log-transformed data constructed with the “psych” package (Revelle, 2017) in the R environment. The panels present scatter plots of variable pairs in which the x axis in each plot is the column variable, and the y axis the row variable. Each scatter plot shows the loess locally fit regression with an elllipse around the mean; the ellipse indicates one standard deviation of the x and y data. The diagonal shows the histogram of the data.
