# Supplementary material for: Geometry, Allometry and Biomechanics of Fern Leaf Petioles: Their Significance for the Evolution of Functional and Ecological Diversity Within the Pteridaceae
Source: Front Plant Sci. 2018 Mar 7;9:197. doi: 10.3389/fpls.2018.00197 (PMC5850050; doi:10.3389/fpls.2018.00197)
Supplement: Supplementary Image 1 — Additional petiolar cross-sections from the surveyed Pteridaceae taxa. [file Image1.pdf]

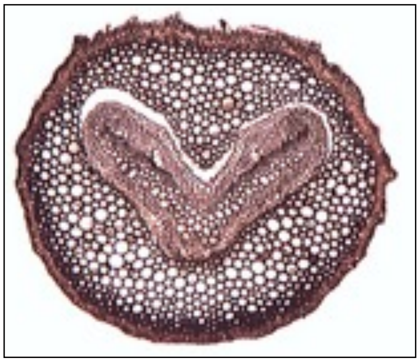

Bommeria hispida

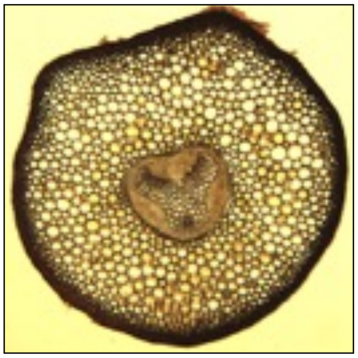

Pellaea truncata

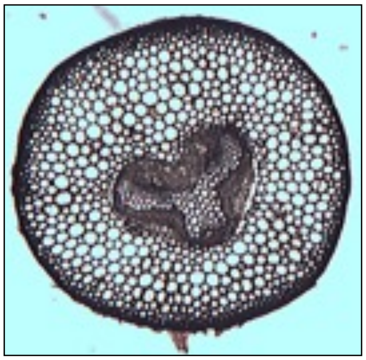

Notholaena standleyi

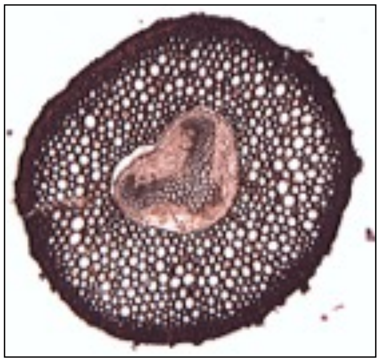

Myriopteris wootonii

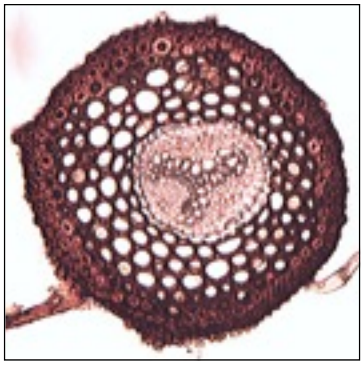

Myriopteris gracilis

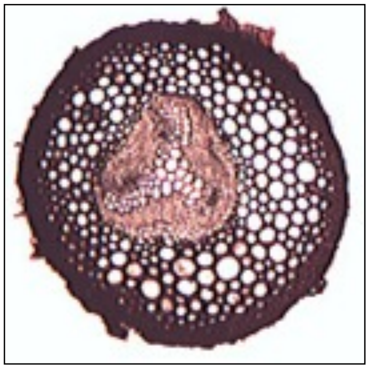

Myriopteris lindheimeri

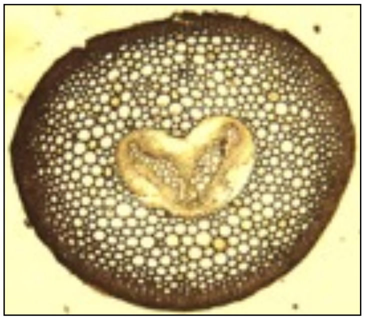

Gaga angustifolia

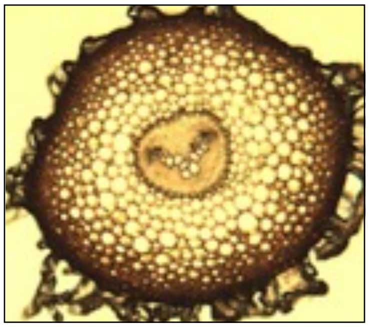

Jamesonia scammaniae

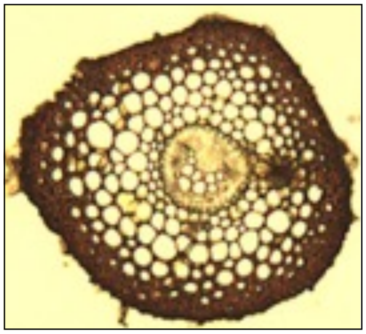

Jamesonia alstonii

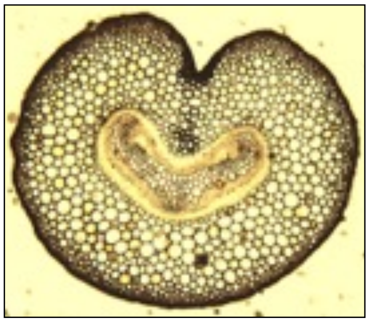

Gaga marginata

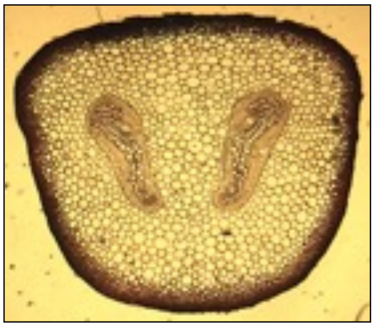

Adiantum latifolium

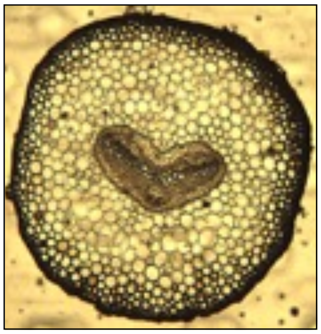

Hemionitis palmata

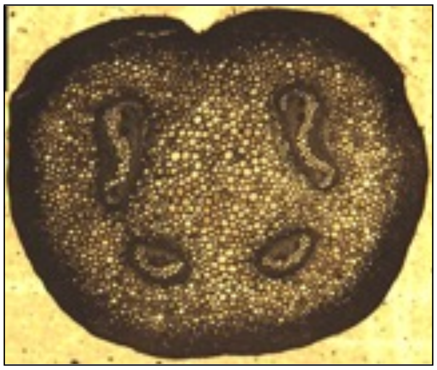

Pityrogramma ebenea

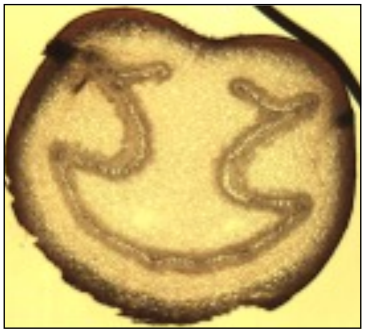

Dennstaedtia cicutaria

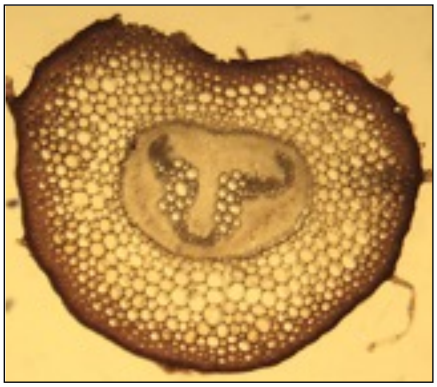

Eriosorus flexuosus

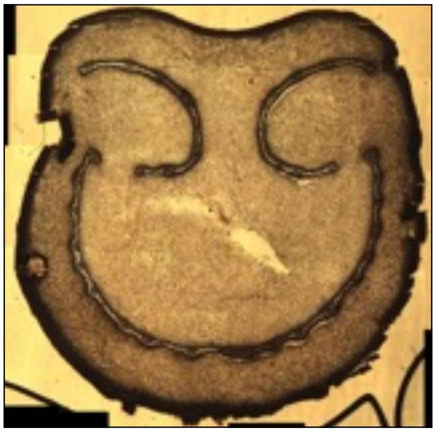

Pteris livida

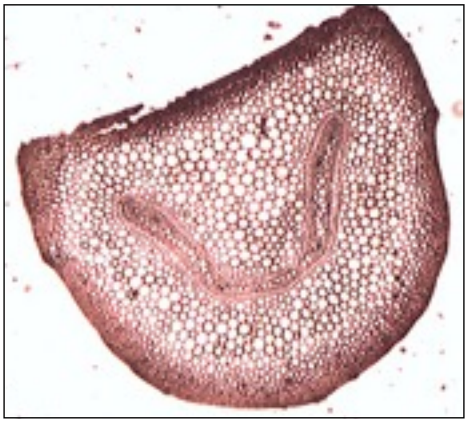

Pteris pungens

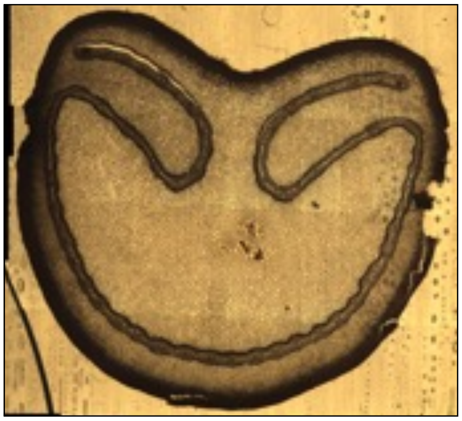

Pteris podophylla

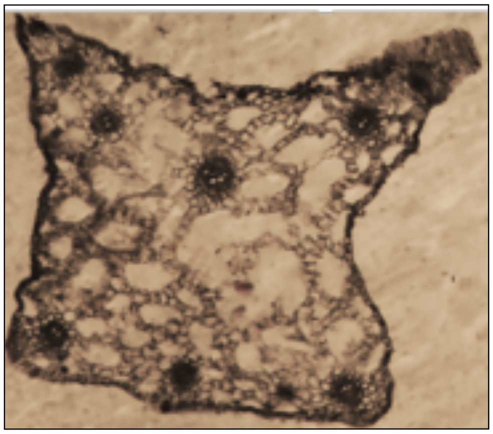

Ceratopteris thalictroides
