# Supplementary figures and images for: Geometry, Allometry and Biomechanics of Fern Leaf Petioles: Their Significance for the Evolution of Functional and Ecological Diversity Within the Pteridaceae
Source: Front Plant Sci. 2018 Mar 7;9:197. doi: 10.3389/fpls.2018.00197 (PMC5850050; doi:10.3389/fpls.2018.00197)

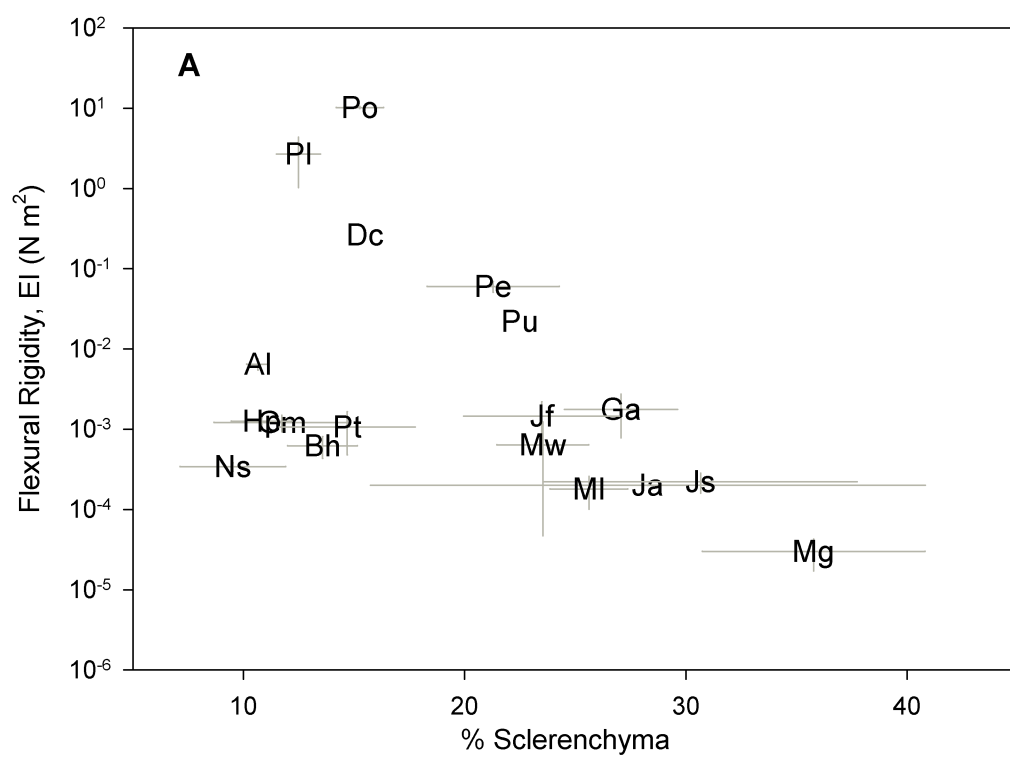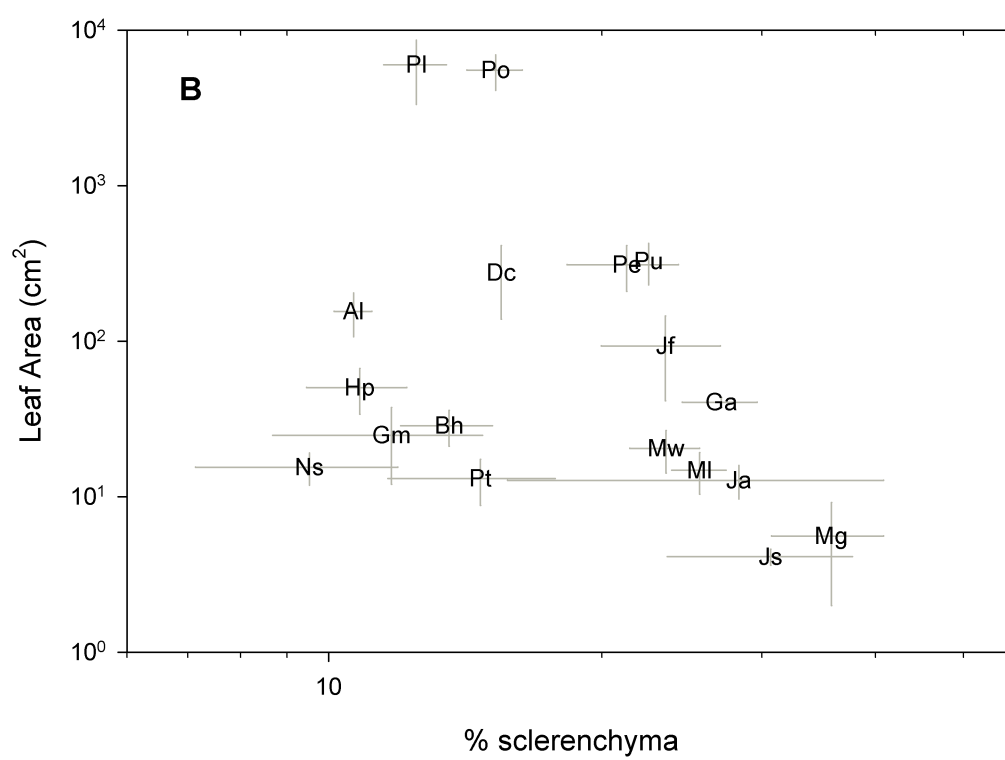

Supplement: Supplementary Image 2 — Non-significant relationships between the percentage of sclerenchyma that occupies the petiole and flexural rigidity (A) and leaf area (B). [file Image2.pdf]

Sterome Traits and Modeled Biomechanics

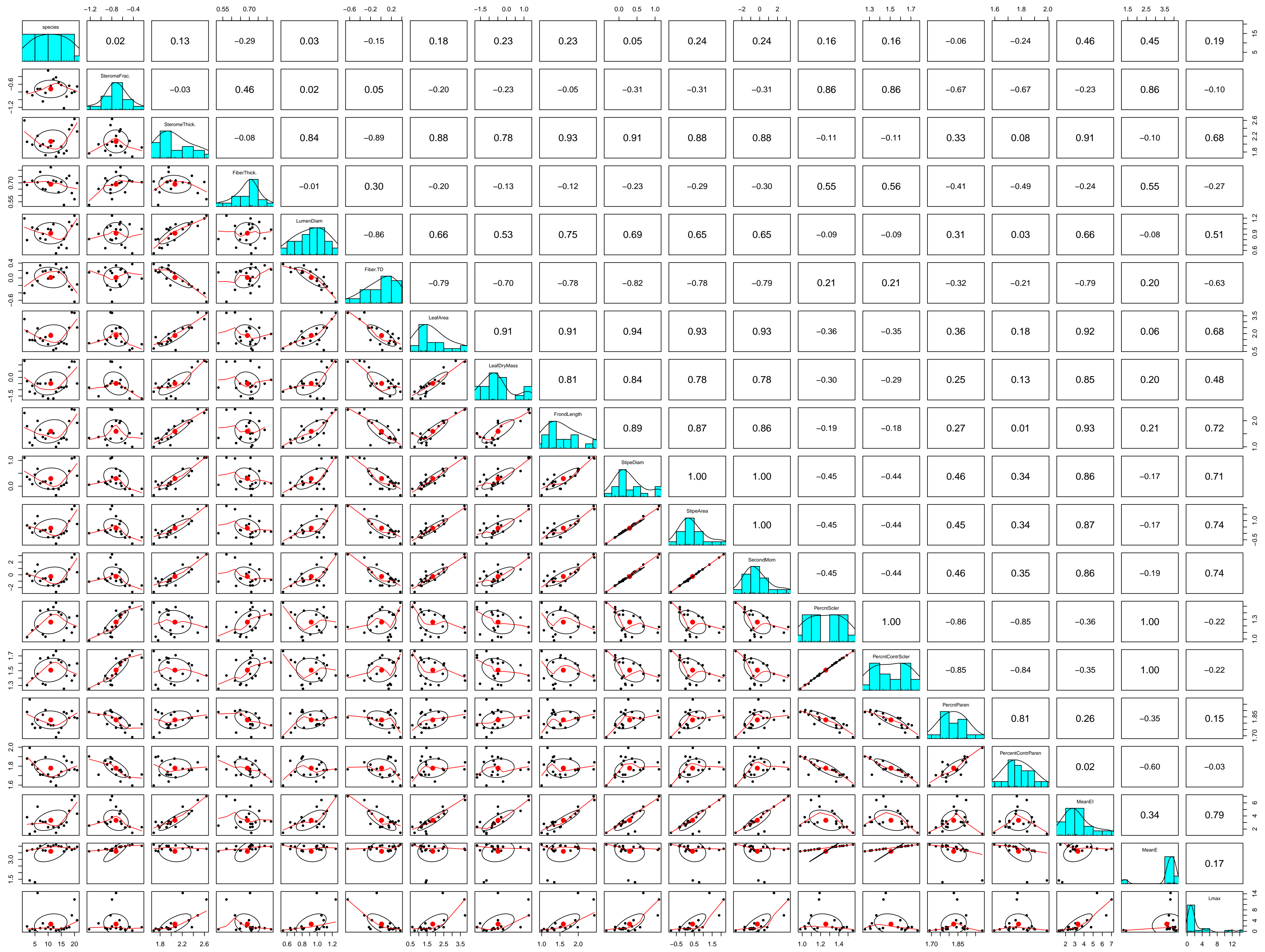

Supplement: Supplementary Data Sheet 2 — A pair-wise correlation matrix of the log-transformed data constructed with the “psych” package (Revelle, 2017) in the R environment. The panels present scatter plots of variable pairs in which the x axis in each plot is the column variable, and the y axis the row variable. Each scatter plot shows the loess locally fit regression with an elllipse around the mean; the ellipse indicates one standard deviation of the x and y data. The diagonal shows the histogram of the data. [file DataSheet2.pdf]
