# Supplementary material for: Geometry, Allometry and Biomechanics of Fern Leaf Petioles: Their Significance for the Evolution of Functional and Ecological Diversity Within the Pteridaceae
Source: Front Plant Sci. 2018 Mar 7;9:197. doi: 10.3389/fpls.2018.00197 (PMC5850050; doi:10.3389/fpls.2018.00197)
Supplement: Supplementary Data Sheet 1 — A list of Pteridaceae species, Genbank atpA and rbcL sequence ID numbers, and source attributions. [file DataSheet1.pdf]

Clade	Taxon Author	Alignments	atpA	atpA reference	Comments
Outgroup	Dennstaedtia cicutaria (Sw.) T.Moore	>Dennstaedtia_cicutaria	EF463652	Schuettpelz and Pryer, 2007	Sequences are from D. dissecta (Sw.) T.Moore
Parkerioideae	Acrostichum aureum L.	>Acrostichum_aureum	JF303991	Kuo et al., 2011	
Parkerioideae	Ceratopteris thalictroides (L.) Brongn.	>Ceratopteris_thalictroides	EF452082	Schuettpelz et al., 2007	Sequences are from C. richardii Brongn.
Pteridoideae	Jamesonia alstonii A.F.Tryon	>Jamesonia_alstonii	KJ416273	Cochran et al., 2014	
Pteridoideae	Jamesonia flexuosa (Kunth) Christenh.	>Jamesonia_flexuosa	KM007562	Zhang et al., 2015	
Pteridoideae	Jamesonia scammaniae A.F.Tryon	>Jamesonia_scammaniae	KM007566	Zhang et al., 2015	
Pteridoideae	Pityrogramma ebenea (L.) Proctor	>Pityrogramma_ebenea	KM007572	Zhang et al., 2015	
Pteridoideae	Pteris livida Mett.	>Pteris_livida	KM007626	Zhang et al., 2015	
Pteridoideae	Pteris podophylla Sw.	>Pteris_podophylla	KM007643	Zhang et al., 2015	
Pteridoideae	Pteris pungens Willd.	>Pteris_pungens	KM007649	Zhang et al., 2015	
Vittarioideae	Adiantum latifolium Lam.	>Adiantum_latifolium	EF452073	Schuettpelz et al., 2007	Sequences are from A. tetraphyllum Humb. & Bonpl. ex Willc
Vittarioideae	Polytaenium citrifolium (L.) Schuettp.	>Polytaenium_citrifolium	EF452075	Schuettpelz et al., 2007	
Cheilanthoideae	Bommeria hispida (Mett. ex Kuhn) Underw.	>Bommeria_hispida	EF452081	Schuettpelz et al., 2007	
Cheilanthoideae	Gaga angustifolia (Kunth) F.W.Li & Windham	>Gaga_angustifolia	JQ855918	Johnson et al., 2012	Sequences are from G. kaulfussii (Kunze) F.W.Li & Windham
Cheilanthoideae	Gaga marginata (Kunth) F.W.Li & Windham	>Gaga_marginata	EU268727	Rothfels et al., 2008	Sequences are from G. arizonica (Maxon) F.W.Li & Windhan
Cheilanthoideae	Hemionitis palmata L.	>Hemionitis_palmata	EF452098	Schuettpelz et al., 2007	
Cheilanthoideae	Myriopteris gracilis Fée	>Myriopteris_gracilis	KF961720	Grusz et al., 2014	
Cheilanthoideae	Myriopteris lindheimeri (Hook.) J.Sm.	>Myriopteris_lindheimeri	KF961735	Grusz et al., 2014	
Cheilanthoideae	Myriopteris wootonii (Maxon) Grusz & Windham	>Myriopteris_wootonii	KF961759	Grusz et al., 2014	
Cheilanthoideae	Notholaena standleyi Maxon	>Notholaena_standleyi	EU268760	Rothfels et al., 2008	
Cheilanthoideae	Pellaea truncata Goodd.	>Pellaea_truncata	EF452110	Schuettpelz et al., 2007	

Clade	Taxon Author	Alignments	rbcL	rbcL reference	Comments
Outgroup	Dennstaedtia cicutaria (Sw.) T.Moore	>Dennstaedtia_cicutaria	EF463166	Schuettpelz and Pryer, 2007	Sequences are from D. dissecta (Sw.) T.Moore
Parkerioideae	Acrostichum aureum L.	>Acrostichum_aureum	KU744801	Zumkeller et al., 2016	
Parkerioideae	Ceratopteris thalictroides (L.) Brongn.	>Ceratopteris_thalictroides	AB059585	Masuyama et al., 2002	Sequences are from C. richardii Brongn.
Pteridoideae	Jamesonia alstonii A.F.Tryon	>Jamesonia_alstonii	KJ416329	Cochran et al., 2014	
Pteridoideae	Jamesonia flexuosa (Kunth) Christenh.	>Jamesonia_flexuosa	KM008132	Zhang et al., 2015	
Pteridoideae	Jamesonia scammaniae A.F.Tryon	>Jamesonia_scammaniae	KM008135	Zhang et al., 2015	
Pteridoideae	Pityrogramma ebenea (L.) Proctor	>Pityrogramma_ebenea	KM008141	Zhang et al., 2015	
Pteridoideae	Pteris livida Mett.	>Pteris_livida	KM008192	Zhang et al., 2015	
Pteridoideae	Pteris podophylla Sw.	>Pteris_podophylla	KM008209	Zhang et al., 2015	
Pteridoideae	Pteris pungens Willd.	>Pteris_pungens	KM008215	Zhang et al., 2015	
Vittarioideae	Adiantum latifolium Lam.	>Adiantum_latifolium	EF452135	Schuettpelz et al., 2007	Sequences are from A. tetraphyllum Humb. & Bonpl. ex Willc
Vittarioideae	Polytaenium citrifolium (L.) Schuettp.	>Polytaenium_citrifolium	KC984523	Rothfels and Schuettpelz, 2014	
Cheilanthoideae	Bommeria hispida (Mett. ex Kuhn) Underw.	>Bommeria_hispida	EF452142	Schuettpelz et al., 2007	
Cheilanthoideae	Gaga angustifolia (Kunth) F.W.Li & Windham	>Gaga_angustifolia	JQ855930	Johnson et al., 2012	Sequences are from G. kaulfussii (Kunze) F.W.Li & Windham
Cheilanthoideae	Gaga marginata (Kunth) F.W.Li & Windham	>Gaga_marginata	EU268776	Rothfels et al., 2008	Sequences are from G. arizonica (Maxon) F.W.Li & Windhan
Cheilanthoideae	Hemionitis palmata L.	>Hemionitis_palmata	KC984525	Rothfels and Schuettpelz, 2014	
Cheilanthoideae	Myriopteris gracilis Fée	>Myriopteris_gracilis	KF961783	Grusz et al., 2014	
Cheilanthoideae	Myriopteris lindheimeri (Hook.) J.Sm.	>Myriopteris_lindheimeri	KF961798	Grusz et al., 2014	
Cheilanthoideae	Myriopteris wootonii (Maxon) Grusz & Windham	>Myriopteris_wootonii	KF961823	Grusz et al., 2014	
Cheilanthoideae	Notholaena standleyi Maxon	>Notholaena_standleyi	EU268805	Rothfels et al., 2008	
Cheilanthoideae	Pellaea truncata Goodd.	>Pellaea_truncata	EF452164	Schuettpelz et al., 2007	
